# Maize metabolome and proteome responses to controlled cold stress partly mimic early‐sowing effects in the field and differ from those of Arabidopsis

**DOI:** 10.1111/pce.13993

**Published:** 2021-01-25

**Authors:** Maria Urrutia, Mélisande Blein‐Nicolas, Sylvain Prigent, Stéphane Bernillon, Catherine Deborde, Thierry Balliau, Mickaël Maucourt, Daniel Jacob, Patricia Ballias, Camille Bénard, Hélène Sellier, Yves Gibon, Catherine Giauffret, Michel Zivy, Annick Moing

**Affiliations:** ^1^ Biologie du Fruit et Pathologie, UMR 1332, Centre INRAE de Nouvelle Aquitaine‐Bordeaux INRAE, Univ. Villenave d'Ornon France; ^2^ INRAE, CNRS, AgroParisTech, GQE‐Le Moulon Univ. Paris‐Saclay Gif‐sur‐Yvette France; ^3^ PAPPSO, doi:10.15454/1.5572393176364355E12, GQE‐Le Moulon Gif‐sur‐Yvette France; ^4^ PMB‐Metabolome, INRAE, 2018, Bordeaux Metabolome, doi:10.15454/1.5572412770331912E12, MetaboHUB, PHENOME, IBVM, Centre INRAE de Nouvelle Aquitaine‐Bordeaux Villenave d'Ornon France; ^5^ INRAE, UE GCIE, Estrées‐Mons Peronne France; ^6^ INRAE, Univ. Liège, Univ. Lille, Univ. Picardie Jules Verne, BioEcoAgro Peronne France; ^7^ Present address: Dtp. Biología Molecular y Bioquímica Univ. Málaga Málaga Spain

**Keywords:** acclimation, chilling, cold stress, low temperature, maize, metabolomics, plant leaf, proteomics

## Abstract

In Northern Europe, sowing maize one‐month earlier than current agricultural practices may lead to moderate chilling damage. However, studies of the metabolic responses to low, non‐freezing, temperatures remain scarce. Here, genetically‐diverse maize hybrids (*Zea mays,* dent inbred lines crossed with a flint inbred line) were cultivated in a growth chamber at optimal temperature and then three decreasing temperatures for 2 days each, as well as in the field. Leaf metabolomic and proteomic profiles were determined. In the growth chamber, 50% of metabolites and 18% of proteins changed between 20 and 16°C. These maize responses, partly differing from those of Arabidopsis to short‐term chilling, were mapped on genome‐wide metabolic maps. Several metabolites and proteins showed similar variation for all temperature decreases: seven MS‐based metabolite signatures and two proteins involved in photosynthesis decreased continuously. Several increasing metabolites or proteins in the growth‐chamber chilling conditions showed similar trends in the early‐sowing field experiment, including *trans*‐aconitate, three hydroxycinnamate derivatives, a benzoxazinoid, a sucrose synthase, lethal leaf‐spot 1 protein, an allene oxide synthase, several glutathione transferases and peroxidases. Hybrid groups based on field biomass were used to search for the metabolite or protein responses differentiating them in growth‐chamber conditions, which could be of interest for breeding.

## Abstract 2

Metabolomic and proteomic responses of diverse maize hybrids to progressive decreasing temperatures were larger from 20 to 16°C, partly mimicked those in the field, and involved primary and specialized metabolisms.

**Abbreviations**
ANOVA: analysis of variance
d_20°C,_ equivalent days at 20°C after emergence
LC‐ESI‐QTOF‐MS: liquid chromatography electrospray‐ionization time‐of‐flight mass spectrometry
NMR: nuclear magnetic resonance spectroscopy
OSC‐PLS‐DA: orthogonal signal correction partial least‐square discriminant analysis
PCA: principal component analysis
PS: photosystem
TCA: tricarboxylic acid
VIP: variable importance in the projection

## 1 INTRODUCTION

Temperature is a crucial factor for plant crop development from germination to harvest and consequently for productivity, especially for cereals which are major sources of human food and animal feed. Among these, maize is a cereal of great economic importance, with a worldwide production of about 1.15 billion tons for grain and 12 million tons for green maize in 2018 (faostat3.fao.org, FAO food and agriculture data). In Northern Europe, early sowing, that is, sowing about one‐month earlier than current agricultural practices, is used to maximize the yield of early‐maturing hybrid maize varieties (Parent et al., 2018; Parker, Shonkwiler, & Aurbacher, 2017). For cold‐tolerant genotypes, early sowing leads to moderate damage of seedlings due to chilling temperatures during the vegetative stages, without any clear visible phenotype in the field. However, for cold‐susceptible genotypes, chilling stress may induce symptoms at plant level such as chlorosis, development and growth retardation, but also injury or even necrosis in shoot or root tissues (Duran‐Garzon et al., 2020; Greaves, 1996). Such symptoms may be associated with reduced photosynthesis and increased reactive oxygen species (ROS) levels, leading possibly to photoinhibition (Foyer, Vanacker, Gomez, & Harbinson, 2002; Szalai et al., 2018). Chilling may also induce changes at the cell level such as osmotic stress, membrane modification and low protein activity. The corresponding metabolic changes go beyond a general reduction of enzyme activities with a major reconfiguration of various metabolic processes (Krasensky & Jonak, 2012). A chilling acclimation process may increase tolerance to low temperatures. During chilling acclimation, reprogramming of gene expression leads to the accumulation of protective proteins and metabolites such as compatible solutes (including soluble sugars, sugar alcohols, proline and betaine) and ROS protectors, during which signalling metabolites are involved (Zhu, Dong, & Zhu, 2007). A better knowledge of the metabolic responses to cold may help accelerate the breeding schemes and the release of new cold‐tolerant maize varieties. Metabolomics and proteomics appear to be the most appropriate methods for this purpose (Rai & Saito, 2016).

Nowadays, most plant metabolomics studies combine several analytical strategies (Alseekh & Fernie, 2018; Hall, 2011). Gas chromatography coupled with mass spectrometry or proton NMR spectroscopy (^1^H‐NMR) of polar extracts provides information about primary metabolites. Liquid chromatography coupled with mass spectrometry (LC–MS) of semi‐polar extracts provides information about specialized metabolites, for example, flavonoids, hydroxycinnamates and benzoxazinoids. Recently, such analytical approaches have been applied widely to cereals (Balmer, Flors, Glauser, & Mauch‐Mani, 2013; Gálvez, 2020; Khakimov, Bak, & Engelsen, 2014; Saia, Fragasso, De Vita, & Beleggia, 2019), including maize for studies on environmental effects (Sun et al., 2016; Tang et al., 2017). Besides primary metabolites (Cañas et al., 2017; Sun, Li, et al., 2016), maize leaf contains several families of specialized metabolites involved in stress responses, such as quinic acid derivatives (Cortés‐Cruz, Snook, & McMullen, 2003), flavone glycoside maysin (Rector, Liang, & Guo, 2003) and benzoxazinoids (Zhou, Richter, & Jander, 2018). Besides metabolomic analysis, mass spectrometry also helps quantify hundreds to thousands of proteins simultaneously, through proteomic approaches in model plant species or crops (Jan, Qazi, Raja, & John, 2019; Jorrín‐Novo et al., 2015), for example, cereals (Pechanova & Pechan, 2017).

Metabolomics or proteomics (Ahmad et al., 2016; Hu, Rampitsch, & Bykova, 2015) have been used extensively to understand plant responses to abiotic stresses including temperature stress (Bocian et al., 2015; Glaubitz, Erban, Kopka, Hincha, & Zuther, 2015; Sun, Gao, Li, Fu, & Zhang, 2016; Thomason et al., 2018; Wang et al., 2016; Zhang et al., 2016; Zhao et al., 2016). For maize leaf, metabolomics shows that response to chilling induced by early sowing involves the primary and the specialized metabolisms, with an accumulation of several biomass precursors, compatible solutes and antioxidant compounds (Lamari et al., 2018). Proteomics analyses confirm the complexity of the response to cold‐induced stress and identify proteins involved in posttranslational modifications, signal transduction, lipid metabolism, inorganic ion transport, amino acid metabolism and carbohydrate and energy metabolism (Wang et al., 2016). The combination of metabolomics and proteomics is now used to understand the fundamentals of stress physiology and biochemistry in crops, including cereals (Chmielewska et al., 2016; Gayen et al., 2019). The mining of metabolomic and proteomic data together with pathway enrichment analyses reveal the underlying physiological and molecular mechanisms induced by stress (Wang et al., 2018). Furthermore, combined mining of metabolomic and proteomic data using Sparse Partial Least Squares (sPLS) and network analyses can reveal molecular interaction networks of positive and negative correlations between proteins and metabolites involved in stress response (Escandon et al., 2017).

The present study used untargeted metabolomic and proteomic approaches on a collection of genetically diverse maize hybrids cultivated in a growth chamber and in the field. It aimed at deciphering the molecular mechanisms involved in the response to progressively decreasing temperatures at the vegetative stage and identifying metabolic responses that could be extrapolated from growth‐chamber conditions to early sowing in the field. Hybrids were also grouped according to their biomass in the field to search for the metabolite, metabolite signature or protein responses differentiating them in growth‐chamber conditions, and which could be of potential interest for proposing ideotypes and breeding purposes.

## 2 MATERIALS AND METHODS

### 2.1 Plant material, growth conditions and sampling

Eighteen genetically diverse dent maize inbred‐lines (*Zea mays* ssp. *mays*, Table 1) from eight admixture groups were selected according to their diversity, based on pedigree and genotyping while keeping close flowering dates (Ganal et al., 2011; Rincent et al., 2014). They were crossed with the UH007 flint inbred‐line (Univ. Hohenheim, Germany), which was developed to improve the combining ability with Iodent and Stiff‐Stalk lines for earliness, yield of grain and stover. The seeds were sown as hybrids, either in a growth chamber or in the field.

**TABLE 1:** List of the 18 maize hybrids obtained from dent panel lines crossed with a flint inbred line. Accession indicates the female inbred common name followed by the origin of the seed lot used for the project. The male tester line was UH007

Accession	Origin^a^	Admixture group^b^	Silage‐earliness group
A374_inra	USDA	Minnesota13	Mid‐Early
B89_inra	USDA	Minnesota13	Mid‐Early
EZ11A_csic	CSIC	Minnesota13	Very Late
MS153_inra	USDA	Minnesota13	Late
Oh02_inra	USDA	Minnesota13	Mid‐Early
Oh33_inra	USDA	Minnesota13	Early
D06_uh	UH	D06family	Very Early
FV353_inra	INRA	Iodent	Early
PH207_usda	USDA	Iodent	Mid‐Early
B100_uh	USDA	LancasterOh43	Late
B97_inra	USDA	Minnesota13	Late
Mo17_inra	USDA	LancasterMo17	Late
B104_inra	USDA	StiffStalkB73	Very Late
B73_inra	USDA	StiffStalkB73	Late
B84_inra	USDA	StiffStalkB73	Late
EC169_ciam	CIAM	StiffStalkB14	Early
F1808_inra	INRA	StiffStalkB14	Mid‐Early
F618_inra	INRA	StiffStalkB14	Mid‐Early

^a^ USDA, United States Department of Agriculture, USA; CSIC, Consejo Superior de Investigaciones Científicas, Spain; UH, Universität Hohenheim, Germany; CIAM, Centro Investigacións Agrarias de Mabegondo, Spain.

^b^ Admixture groups are based on Panzea SNPs from Illumina MaizeSNP50 BeadChip (Ganal et al., 2011; Rincent et al., 2014).

For the growth‐chamber experiment, the experimental design consisted in four temperatures (20, 16, 13 and 8.5°C settings) with three steps of decreasing temperatures lasting for 2 days each. The corresponding temperature data were recorded and means were calculated. For space issues, nine successive experiments were necessary to obtain enough plant material and representative sampling for three replicates (Table S1). Each experiment comprised all hybrids with eight plants per hybrid. Two plants were devoted to physiological measurements and the six other were used for leaf sampling that was randomized to avoid confounding effects due to circadian changes. Seeds were germinated for 4 days at 20°C in the dark between two wet blotting papers and transferred into 1.5‐L pots filled with TS3‐607 potting substrate (Klasmann‐Deilmann, Bourgoin Jallieu, France). For each hybrid, a total of 12 seeds were sown in four pots, each pot containing three seeds that were thinned to two plants after emergence. Plants were cultivated with 16‐hr daylight / 8‐hr night, 500 μmol photons m^−2^ s^−1^ photosynthetically active radiation (PAR) at the canopy level, at 20°C (day and night, optimal temperature) for 3 weeks before decreasing the temperature progressively. Samples were taken on the youngest ligulated leaf (the fourth or the fifth leaf) between 10:00 a.m. (4 hr after the beginning of day) and 01:00 p.m. Two central 5‐cm sections were taken on both sides of the main vein for metabolomics and proteomics experiments and immediately frozen in liquid nitrogen. Leaf samples obtained from one to three experiments were pooled to obtain three biological replicates for each genotype‐treatment combination (Table S1). Air temperature was recorded every 5 min at the canopy level and day and night mean temperatures were calculated for each step (Table 2).

**TABLE 2:** Leaf stage and leaf chlorophyll fluorescence parameters of 18 maize hybrids (dent maize inbred lines crossed with flint inbred line UH007) at four temperature steps in growth chamber. Mean ± *SD* (*n* = 18) calculated from mean of three biological replicates per temperature step (except for MO17 at 20°C with two replicates). For fluorescence data, means accompanied by same letter are not significantly different according to Tukey's studentized test (*p* < .05)

Temperature	Measured air temperature (°C)^b^		Ligulated leaf	Visible leaf	Fv/Fm^c^	ΦPSII^c^	ΦPSII measurement
step^a^	Day	Night	number	number			temperature (°C)^d^
20°C	19.4	18.4	4.02 ± 0.08	7.00 ± 0.33	0.78 ± 0.01 a	0.59 ± 0.03 a	21.6 ± 0.4
16°C	14.9	14.0	4.09 ± 0.14	7.39 ± 0.30	0.73 ± 0.02 b	0.34 ± 0.02 b	17.9 ± 0.2
13°C	11.5	10.1	4.26 ± 0.24	7.64 ± 0.31	0.69 ± 0.02 c	0.27 ± 0.01 c	15.1 ± 0.2
8.5°C	6.9	5.6	4.38 ± 0.24	7.73 ± 0.31	0.50 ± 0.03 d	0.13 ± 0.02 d	11.8 ± 0.3

^a^ Growth chamber air temperature.

^b^ Temperature measured at leaf level.

^c^ Leaf chlorophyll fluorescence parameters are operating quantum efficiency of photosystem II (PSII) photochemistry (ΦPSII) and maximum quantum efficiency of PSII (Fv/Fm).

^d^ Temperature recorded at leaf level during measurements with the fluorometer.

A field experiment was performed with 18 hybrids (of which 16 were common with the growth‐chamber experiment) cultivated in two conditions as described by Lamari et al. (2018) with a three‐block design. Early sowing was performed on April 8th 2014 and normal sowing on May 6th 2014. Air temperatures were measured and cumulative thermal times were calculated as the equivalent number of days at 20°C after emergence for each sowing condition (Parent & Tardieu, 2012). Plants in the early‐sowing condition experienced about 2°C‐colder temperatures than plants in the normal‐sowing condition (Figure S1), with a mean temperature of 13.3°C compared to 15.6°C between emergence and leaf harvest. Samples for metabolomics and proteomics were collected on the youngest ligulated leaf (usually the fifth leaf) between 10:30 a.m. and 02:30 p.m. on June 6th 2014 for early sowing and on June 17th 2014 for normal sowing. The experiment design consisted in three blocks harvested in parallel during the harvest duration, with randomization within each block to avoid confounding effects due to circadian changes. For metabolome analyses, central 5‐cm sections without the main vein were sampled on 10 plants per block and bulked to constitute a biological replicate (about 2 g). For proteome analyses, two 6‐mm disks were taken on both sides of the main vein from the central part of the leaf blades of five plants per block. Snap‐frozen leaf samples were stored at −80°C until grinding in liquid nitrogen (2010 Geno/Grinder, Spex, Stanmore, UK). Ground samples were stored at −80°C until proteomic analyses or until they were freeze‐dried. Lyophilized samples were kept at −20°C in dry atmosphere until metabolomic analyses.

### 2.2 Plant phenotyping

In the growth chamber, the leaf number was recorded on the two plants dedicated to physiological measurements per genotype and experiment, just before each leaf sampling time. Chlorophyll fluorescence measurements were performed on the youngest ligulated leaf of the same plants using a pulse amplitude modulated fluorometer (MINI‐PAM, Heinz Walz GmbH, Effeltrich, Germany). The operating quantum yield efficiency of photosystem II (ΦPSII) was measured at a photon flux density of 500 μmol m^−2^ s^−1^. The maximum quantum efficiency of photosystem II (Fv/Fm) was measured after a minimum of 30 min of dark adaptation using leaf clips, to check for the occurrence of photoinhibition (Maxwell & Johnson, 2000). Finally, the visual aspect of plants was monitored with aerial pictures (Figure S2).

### 2.3 ^1^H‐NMR analysis of polar metabolites

For NMR profiling analyses of leaf samples harvested in the growth chamber or in the field, polar metabolites were extracted from 20 ± 1 mg dry weight (DW) using a hot ethanol/water series and quantified by ^1^H‐NMR as previously described (Lamari et al., 2018). Special care was taken to allow absolute quantification of individual metabolites, including the use of a 90° pulse angle and an electronic reference with calibration curves for quantification. The assignments of 26 metabolites in the NMR spectra were based on Lamari et al. (2018). The NMR spectra were deposited in the Data INRAE repository (https://data.inrae.fr/dataverse/MaizeChill, doi:10.15454/F2CZ1R).

### 2.4 Starch and protein measurements

Starch content of the leaf samples was determined enzymatically in the previously obtained pellets after polar compound extraction (Hendriks, Kolbe, Gibon, Stitt, & Geigenberger, 2003), using 96‐well polystyrene microplates and expressed in glucose equivalents as described in Lamari et al. (2018). Total protein content was determined colorimetrically in the pellet resuspended in 100 mM NaOH (Bradford, 1976). Starch and total proteins were expressed on a DW basis.

### 2.5 LC‐ESI‐QTOF‐MS analysis of semi‐polar metabolites

Lyophilized maize samples (10 ± 0.3 mg DW) were extracted with 1 ml of methanol/water (70/30, v/v) with 0.1% formic acid and methyl vanillate as internal standard. Methanolic extracts were analysed by LC‐QTOF‐MS as detailed previously (Lamari et al., 2018). The data were processed using XCMS [RRID:SCR_015538, (Smith, Want, O'Maille, Abagyan, & Siuzdak, 2006)]. Methyl vanillate intensity was used to check whether the injection was performed correctly. Variable intensities were corrected for technical drift with a “Quality Control” sample (mix of all samples) injected every 10 samples. Blank extractions were used to remove contaminant features. For the growth‐chamber sample set, this resulted in a high‐quality dataset retaining 1,375 ions over the 1,675 initial ones and used for further statistical analyses. Molecular formulae were generated using the SmartFormula software (Bruker, Bremen, Germany). Initial annotations were based on previously published data (Lamari et al., 2018). Complementary annotation work and putative name assignments (Table S2) were also achieved by comparing with MS‐related information in the literature (Zhou et al., 2019) and databases (MassBank RRID:SCR_015535, MoNA RRID:SCR_015536, mzCloud RRID:SCR_014669). For the field samples, LC‐QTOF‐MS acquisitions were performed in another batch and processed apart. The correspondence between variables of the two sets was based on retention time and accurate *m/z*. The LC–MS data and metadata were deposited in the Data INRAE repository (https://data.inrae.fr/dataverse/MaizeChill/, doi:10.15454/J9KO72).

### 2.6 LC–MS/MS shotgun proteomics

Proteins were extracted from 50 mg of fresh material of leaf samples. Protein extraction and digestion were performed as described previously (Blein‐Nicolas et al., 2020). LC–MS/MS analyses of protein digests (400 ng of peptides) were performed using a NanoLC‐Ultra System (nano2DUltra, Eksi‐gent, Les Ulis, France) connected to a Q‐Exactive mass spectrometer (Thermo Electron, Waltham, MA) as described previously (Balliau, Blein‐Nicolas, & Zivy, 2018), except for the linear gradient which was shortened to 40 min, and for the mass range for Full MS scan which was extended to 350–1,400 m/z. Raw datafiles were transformed into mzXML open source format, using msconvert software in the ProteoWizard 3.0.3706 package (Kessner, Chambers, Burke, Agus, & Mallick, 2008). During conversion, MS and MS/MS data were centroided. Peptide and protein identification were performed with X!Tandem software ([Craig & Beavis, 2004], version Alanine 2017.2.1.4), using the maize genome (http://www.maizesequence.org/ version 5a) and a home‐made contaminant database. Protein inference was performed using X!TandemPipeline (Langella et al., 2017) with the following parameters: at least two peptides per protein, peptide e‐value <0.01, protein e‐value <10^−5^. The false discovery rate (FDR) was assessed by searching a decoy database and estimated at 0.07% for peptides and 0% for proteins. Peptide ions were quantified based on the extracted ion chromatograms (XIC), using MassChroQ software version 2.2 (Valot, Langella, Nano, & Zivy, 2011). Their intensity was normalized as described previously (Millan‐Oropeza et al., 2017). Proteins represented by at least two reproducible and consistent peptides were quantified by summing their intensities as in Balliau et al. (2018), in order to measure their relative abundance. For the growth‐chamber experiment, the absolute content of each protein on a DW basis was also estimated using its relative abundance and the total protein content of each sample. Protein annotations were extracted from maizegdb (https://www.maizegdb.org/, RRID:SCR_006600, (Portwood II et al., 2018)) and complemented using enzymatic data included in CornCyc v8.0 (https://www.plantcyc.org/databases/corncyc/8.0).

The mass spectrometry proteomics data were deposited to the ProteomeXchange Consortium via the PRIDE (Perez‐Riverol et al., 2018) partner repository with the dataset identifiers PXD021790 and PXD021754.

### 2.7 Data analyses

For chlorophyll fluorescence measurements, ANOVAs were performed after fitting a linear mixed model including genotype, temperature and their interaction as fixed effects and experiment as random effect, using the lmer function in the lmerTest R package. Tukey's test (*p* < .05) was performed with BioStatFlow web application (biostatflow.org, v2.9, (Jacob, Deborde, & Moing, 2020)). Absolute or relative contents of metabolites and starch expressed on a DW basis, and relative abundance of individual proteins were used for statistical analyses, except for volcano plots of proteome data for which both relative abundance and absolute contents were used. Principal component analyses (PCA, correlation matrix), two‐way ANOVAs for genotype, treatment and their interaction (log2‐transformed data for metabolome and log10‐transformed data for proteome data, *p* < .05 with Benjamini‐Hochberg correction for multiple testing [Benjamini & Hochberg, 1995]) and volcano plot analyses (Wilcoxon test, *p* < .01 with Benjamini‐Hochberg correction, with 1.2 ratio between means) were performed using R scripts in BioStatFlow web application (biostatflow.org, v2.8 or v2.9). K‐means clustering of identified compounds and annotated proteins was performed with MultiExperiment Viewer (RRID:SCR_001915, version 4.9) on the means of the 18 hybrids per temperature step (data mean centred and reduced to unit variance, Pearson correlation distance) after volcano plot filtering. For proteome data, function identification and enrichment were performed by assigning proteins to categorical BINs, using Mercator4 (RRID: SRC_014493, v2.0) and studying those BINs using MapMan4 [RRID: SRC_003543, (Schwacke et al., 2019, Usadel et al., 2009)]. Each metabolite was identified using its unique Chemical Entities of Biological Interest identifier (ChEBI ID, https://www.ebi.ac.uk/chebi/, RRID:SCR_002088). Mapping of metabolites and enzymes on metabolic maps was performed using Corncyc v8.0 [https://www.plantcyc.org/databases/corncyc/8.0, RRID: SRC_002110, (Schläpfer et al., 2017)] and MetExplore [https://metexplore.toulouse.inra.fr/, v2.26 and Viz v3.0, (Cottret et al., 2018)]. Orthogonal signal correction partial least‐squares discriminant analyses (OSC‐PLS‐DA) were performed with BioStatFlow to link biomass changes to metabolome or proteome changes after hybrid classification, based on biomass field data. Variable importance in the projection (VIP) scores were used to highlight discriminant variables.

## 3 RESULTS

### 3.1 Short‐term low temperature affects leaf photosynthetic apparatus

At the time of 20°C sample harvest in the growth chamber, the plants had 7.0 visible leaves with 4.0 ligulated ones on average. At the end of the experiment, the plants had 7.7 visible leaves with 4.4 ligulated ones on average, indicating that growth was very limited during the 6 days of cold treatment (Table 2). The comparison of the visual aspect of plants before and after the temperature treatment showed almost no changes between 16°C and 8.5°C for all hybrids (Figure S2).

Photosynthetic performance in the growth chamber was estimated using measurements of chlorophyll fluorescence ((Maxwell & Johnson, 2000), Table 2). PSII maximal efficiency (Fv/Fm) was significantly affected by genotype and temperature effects (ANOVA, *p* < .001) but not by their interaction (*p* > .05). PSII operating efficiency (ϕPSII) was significantly affected by genotype and temperature effects and by their interaction (ANOVA, *p* < .001, *p* < .001 and *p* < .02, respectively). Both the maximal and the operating efficiencies significantly and progressively decreased at each temperature step from 0.78 to 0.50 and from 0.59 to 0.13, respectively (Table 2, Tukey's test *p* < .05).

### 3.2 Most leaf metabolites and proteins are affected by temperature steps in the growth‐chamber experiment

In the growth‐chamber experiment, in addition to starch (Table S3), the metabolome analyses provided the quantification of 34 NMR‐based variables and 1,375 LC–MS‐based variables and the proteome analysis of 1,159 proteins. Two‐way ANOVAs showed that all 1,410 compound variables and 94% of the proteins (1,093 variables, relative abundance) were significantly affected by genotype, temperature or genotype × temperature interaction effects (*p* < .05 after correction for multiple testing, Figure S3). Most compounds and proteins (79.9% and 66.5%, respectively) were affected by both temperature and genotype effects. The genotype × temperature interaction was significant for one metabolite and one protein only: an MS‐based metabolite signature with a retention time of 811 s and an accurate *m*/*z* of 403.1051, and a ubiquinone reductase. Of note, leaf total protein content was significantly affected by the temperature but not by the genotype or the genotype × temperature interaction (two‐factor ANOVA, *p* < .05): it significantly decreased between 20°C and 16°C, and then remained stable at 13°C and 8.5°C and similar to that of both sowing conditions of the field experiment (Table 3, Tukey's test, *p* < .05). Leaf starch content was significantly affected by the temperature and the genotype but not by the genotype × temperature interaction (two‐factor ANOVA, *p* < .05): it significantly increased between 20°C and 16°C, and remained stable at 13°C and 8.5°C and similar to that of both sowing conditions in the field (Table S3, Tukey's test, *p* < .05). We compared the genotype and temperature effects for compounds and proteins using the distributions of their *R* ^*2*^ (Figure S4). On average, the *R* ^*2*^ was higher for the genotype effect than for the temperature or interaction effects for both metabolomic (plus starch) and proteomic data. In addition, the mean *R* ^*2*^ of genotype effect was higher for metabolomic than for proteomic data (0.42 vs. 0.27, respectively), while that of temperature effect was similar (0.13 vs. 0.14, respectively). For a given effect, the *R* ^2^ distribution patterns followed similar trends for compound and for protein data. We thereafter focused on the temperature effects.

**TABLE 3:** Total protein contents in maize leaf for four temperature conditions in growth chamber and field. Mean of 18 hybrid genotypes ± *SD* in growth chamber and 16 in field. Within each experiment, means accompanied by same letter are not significantly different, according to Tukey's studentized test (*p* < .05)

Condition	Protein content (mg/g DW)
Growth‐chamber temperature step	
20°C	140.4 ± 16.2 a
16°C	115.6 ± 9.2 b
13°C	108.3 ± 14.2 b
8.5°C	106.3 ± 11.1 b
Field condition	
Normal sowing	109.2 ± 17.8 a
Early sowing	103.7 ± 19.5 a

### 3.3 Metabolite and protein changes occur mostly between 20°C and 16°C and to a lesser extent at the following temperature steps

**FIGURE 1:**
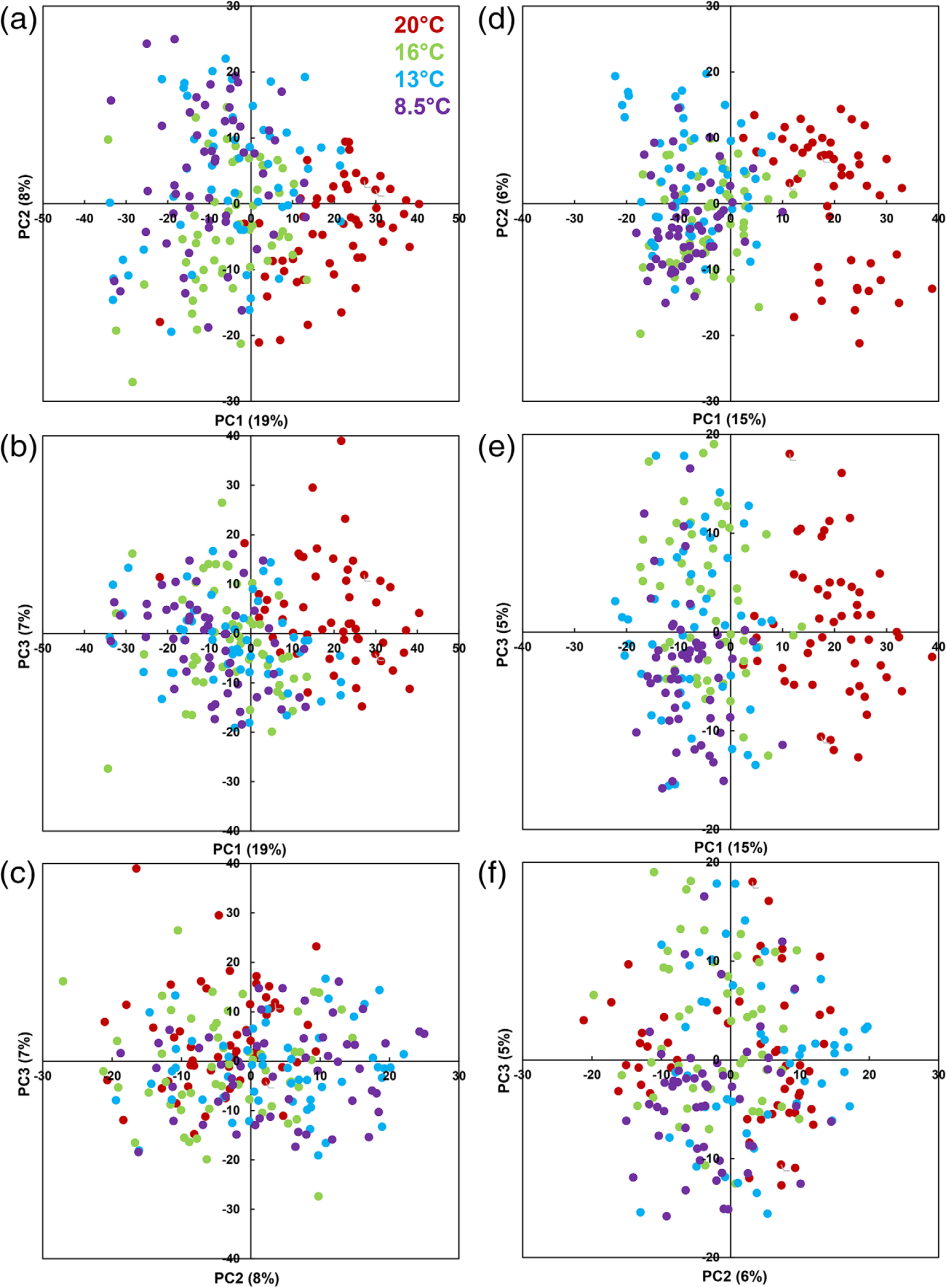
Principal component analysis of metabolome or proteome data measured in young leaf of 18 maize hybrids in the normal (20°C, red symbols) and low temperatures steps (16°C, green, 13°C, blue; 8.5°C, purple) in growth chamber. (a)–(c) PCA scores plot of 1,409 metabolites or metabolite features and starch. (d)–(f) PCA scores plot of 1,159 proteins. (a, d) PC1 × PC2 plan. (b,e) PC1 × PC3 plan. (c, f) PC2 × PC3 plan [Colour figure can be viewed at wileyonlinelibrary.com]

To visualize the compositional changes that occurred in the leaves during the cold experiment, we performed PCAs based on starch and metabolomic data on one hand (Figure 1a–c), and on proteomic data on the other hand (Figures 1d–f). This revealed that both the leaf metabolome and proteome were more strongly affected by the first temperature step (20°C to 16°C) than by the two following ones (PC1, 19% and 15% of the total variance, respectively, Figures 1a, d). The other temperatures could not be separated along the first three PCs, representing in total 33% and 27% of the total variance for metabolome and proteome, respectively.

**FIGURE 2:**
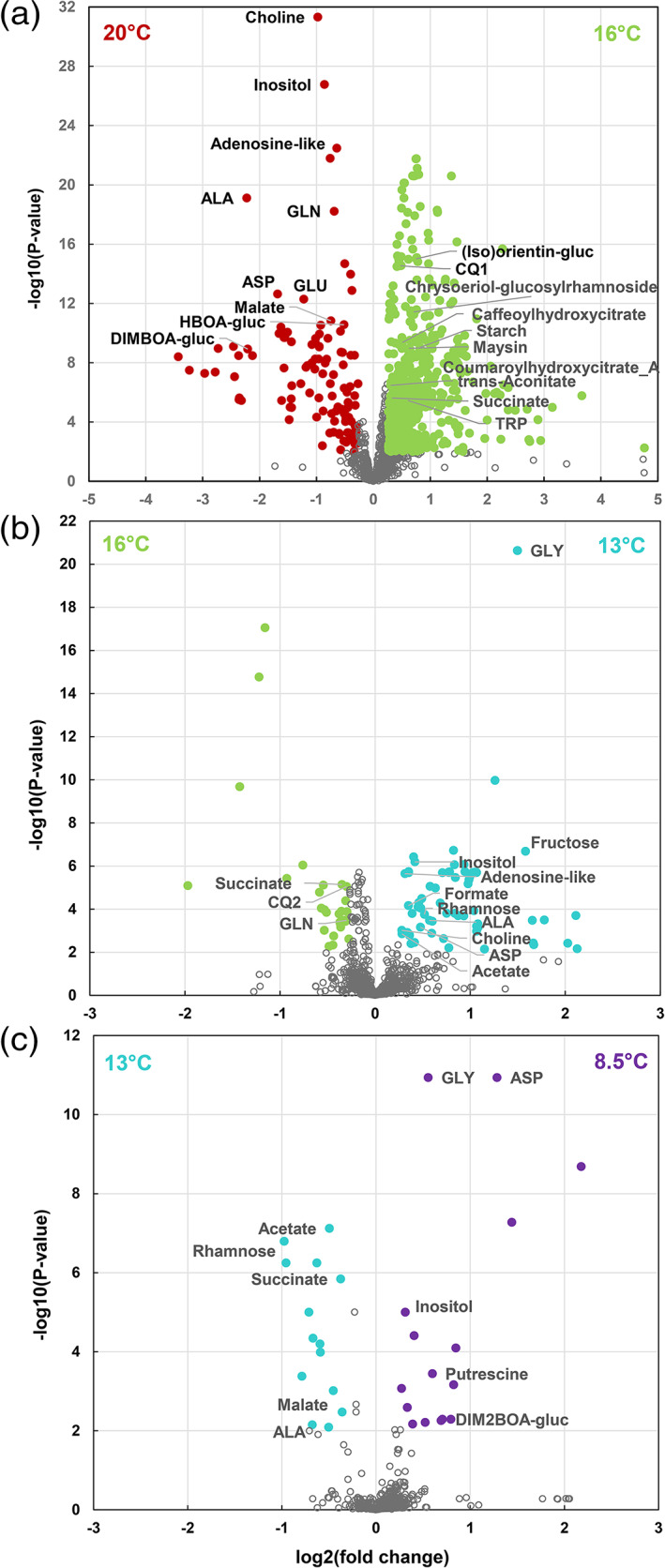
Volcano plot analyses of leaf metabolome and starch data for three temperature‐steps for 18 maize hybrids cultivated in growth chamber (results are detailed in Table S4). Wilcoxon test (*p* < .01 with FDR correction) with thresholds of 0.83 and 1.2 for ratios between means. (a) Changes between 20°C and 16°C. (b) Changes between 16°C and 13°C. (c) Changes between 13°C and 8.5°C. When there are more than 10 identified compounds up or down‐regulated, only the first 10 ones with the lowest *p*‐values are annotated on the volcano plot. gluc, glucoside [Colour figure can be viewed at wileyonlinelibrary.com]

**FIGURE 3:**
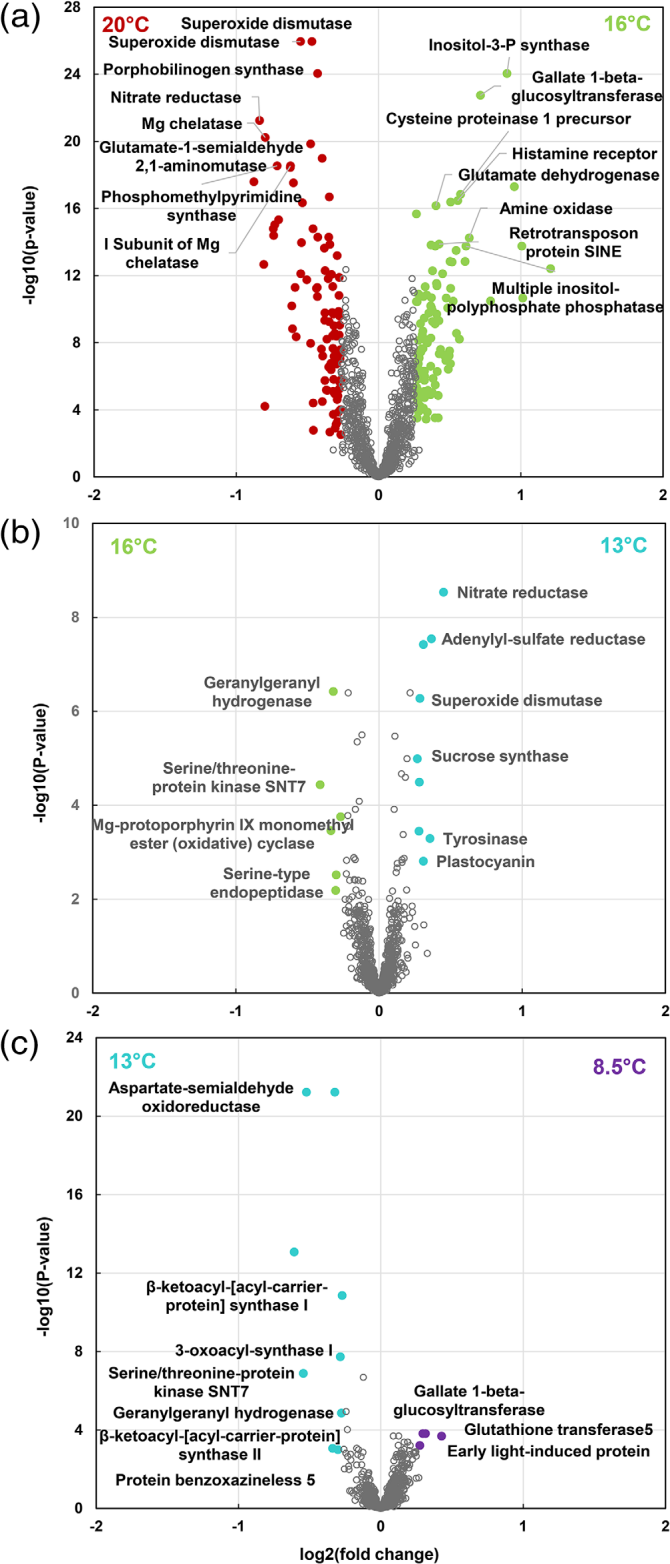
Volcano plot analyses of leaf proteome data (expressed on a relative abundance basis) for three temperature‐steps for 18 maize hybrids cultivated in growth chamber (results and functional annotations are detailed in Table S5). Wilcoxon test (*p* < .01 with FDR correction) with thresholds of 0.83 and 1.2 for ratios between means. (a) Changes between 20°C and 16°C. (b) Changes between 16°C and 13°C. (c) Changes between 13°C and 8.5°C. When there are more than 8 annotated proteins up or down‐regulated, only the first 8 ones with the lowest *p*‐values are annotated on the volcano plot [Colour figure can be viewed at wileyonlinelibrary.com]

To highlight the molecular functions affected at each decreasing temperature step, we then performed a volcano plot analysis separately for metabolome plus starch (1,410 variables including a total of 64 identified compounds, Table S4, Figure 2) and proteome data (1,159 variables including a total of 538 identified proteins, Table S5, Figure 3). When all temperature steps were considered, starch and a total of 716 unique metabolites or metabolite signatures and 214 proteins were affected (corrected *p*‐value <.01, fold change >1.2 Figures 2 and 3, Tables S4, S5). Overall, the numbers of compounds and metabolite signatures or proteins affected decreased from the first to the last temperature step. The number of down‐regulated (resp. up‐regulated) compounds and metabolite signatures were 115, 29, 14 (resp. 586, 66, 16) when temperature changed from 20°C to 16°C, from 16°C to 13°C and from 13°C to 8.5°C, respectively (Figure 2). Among these, 14, 3 and 5 compounds (resp. 23, 11 and 5) were identified. Similarly, the number of down‐regulated (resp. up‐regulated) proteins were 93, 6, 13 (resp. 114, 9, 8) when temperature changed from 20°C to 16°C, from 16°C to 13°C and from 13°C to 8.5°C, respectively (Figure 3). Among these, 59, 4 and 7 proteins (resp. 77, 6 and 3) were annotated. Furthermore, the significance levels and the magnitude of the abundance ratios between means also decreased with temperature.

The molecular functions affected by the temperature decrease were highly dependent on the step considered. Between 20°C and 16°C, the identified down‐regulated metabolites were two sugars (fructose, inositol), six amino compounds (choline, alanine, glutamine, aspartate, glutamate, putrescine), one organic acid (malate) and four benzoxazinoids (DIMBOA‐glucoside, DIM_2_BOA‐glucoside, HBOA‐glucoside, HMBOA‐glucoside) (Figure 2a). Regarding proteins, those whose relative abundances were down‐regulated were mainly involved in protein biosynthesis, coenzyme metabolism and amino acid metabolism (Figure S5). Among the 93 proteins that were down‐regulated at 16°C, the 12 proteins of known function with the lowest *p*‐values were two superoxide dismutases, a porphobilinogen synthase, a nitrate reductase, a magnesium chelatase, a magnesium chelatase subunit, a glutamate‐1‐semialdehyde 2,1‐aminomutase, a phosphomethylpyrimidine synthase, a magnesium‐protoporphyrin IX chelatase, a 30S ribosomal protein and a uroporphyrinogen decarboxylase (Figure 3a). The identified up‐regulated compounds were starch and three sugars (sucrose, rhamnose and raffinose), four organic acids (*trans*‐aconitate, succinate, acetate, shikimate), one amino acid (tryptophan), one benzoxazinoid (DIBOA‐glucoside), four flavonoids ((iso)orientin‐glucoside, chrysoeriol‐glucosylrhamnoside, maysin, (iso)vitexin B) and nine hydroxycinnamic acids (four caffeoylquinates with possible redundancy between the NMR and LC–MS based variables, a coumaroylquinate, a feruloylquinate, a coumaroylhydroxycitrate, a feruloylhydroxycitrate, a caffeoylhydroxycitrate) (Figure 2a). Up‐regulated proteins were mainly involved in protein homeostasis, carbohydrate metabolism, protein modifications and lipid metabolism (Figure S5). Among the 114 proteins that were up‐regulated at 16°C, the 12 proteins of known function with the lowest *p*‐values were an inositol‐3‐phosphate synthase, a gallate β‐glucosyltransferase, a cysteine proteinase precursor, a histamine receptor, a glutamate dehydrogenase, an amine oxidase, a retrotransposon protein, a multiple inositol‐polyphosphate phosphatase, a V‐type ATPase, a plastid‐lipid‐associated protein, lethal leaf‐spot 1 and an early light‐induced protein (Figure 3a). Between 16°C and 13°C, the identified down‐regulated metabolites were succinate, glutamine and a caffeoylquinate. Those that were up‐regulated were three sugars or sugar‐alcohols (fructose, inositol, rhamnose), two organic acids (acetate, formate), five amino compounds (glycine, aspartate, alanine, choline, putrescine) and an adenosine‐like compound (Figure 2b). Six proteins were down‐regulated including a geranylgeranyl hydrogenase, serine/threonine‐protein kinase SNT7, a magnesium‐protoporphyrin IX monomethyl ester (oxidative) cyclase and a serine‐type endopeptidase. Nine proteins were up‐regulated including a nitrate reductase, an adenylyl‐sulfate reductase, a sucrose synthase, a tyrosinase and a plastocyanin (Figure 3b).

Between 13°C and 8.5°C, one sugar (rhamnose), three organic acids (acetate, succinate, malate) and one amino acid (alanine) were decreased. One sugar‐alcohol (inositol), three amino compounds (glycine, aspartate, putrescine) and a benzoxazinoid (DIM_2_BOA‐glucoside) were increased (Figure 2c). Similarly, a small number of proteins were affected (Figure 3c). Nine proteins were down‐regulated, including an aspartate‐semialdehyde oxidoreductase, serine/threonine‐protein kinase SNT7, a β‐ketoacyl‐acyl‐carrier‐protein synthase, a 3‐oxoacyl‐synthase, a geranylgeranyl hydrogenase and protein benzoxazineless 5. Four proteins were up‐regulated, including a gallate‐β‐glucosyltransferase, a glutathione transferase and early light‐induced protein.

Only eight metabolites (Table S4) and two proteins (Table S5) were continuously and significantly up‐ or down‐regulated at each temperature step. Among these, seven MS‐based metabolite signatures decreased continuously, while one MS‐based signature increased continuously. Tentative annotation revealed that all these signatures belonged to four metabolites, of which two were isomers (Table S6). This allowed us to propose most probable elemental formulae but no valid annotation. The two proteins that decreased continuously with the temperature were an SNT7 protein serine/threonine kinase and a geranylgeranyl hydrogenase.

Volcano plot analyses of individual proteins expressed on a DW basis using total protein contents (Table S7), confirmed that most proteins with relative contents down‐regulated with decreasing temperatures followed the same pattern for absolute contents as expected. In addition, they highlighted proteins up‐regulated with decreasing temperatures when expressed in relative abundance but also absolute content (Figure S6) despite decreases in total protein contents: 16 proteins between 16°C and 20°C including an inositol‐3‐P synthase, an ACC oxidase, a gallate 1‐β‐glucosyltransferase, an early light‐induced protein, a V‐type ATPase, an amine oxidase, a multiple inositol‐polyphosphate phosphatase, an histamine receptor, and a nitrate reductase between 16°C and 13°C.

### 3.4 Combining and mapping metabolome and proteome data for growth‐chamber experiment reveal a threshold or progressive response pattern

**FIGURE 4:**
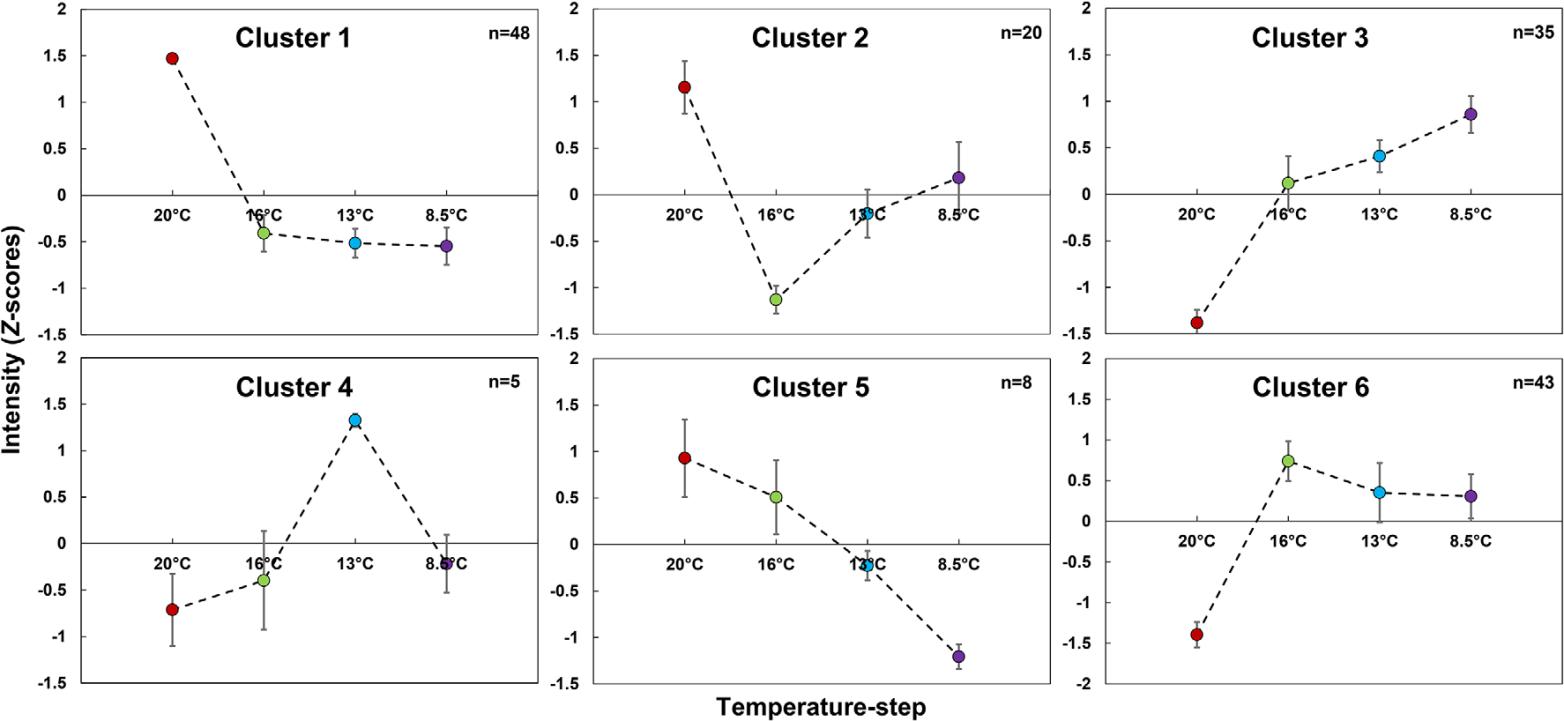
Classification of the patterns of changes in leaf at different temperatures in growth chamber for all identified compounds and annotated proteins selected from volcano plot analyses presented Figures 2 and 3. Clustering of 41 compounds and 118 proteins into six clusters based on K‐means clustering of the means of 18 maize hybrids per temperature step. n indicates the number of variables in each cluster. Compounds or proteins of each cluster are detailed in Table S8 [Colour figure can be viewed at wileyonlinelibrary.com]

The means of all genotypes per temperature step in the growth‐chamber experiment were calculated for starch and for the 40 variables corresponding to identified metabolites, as well as for the 118 annotated proteins highlighted on the volcano plots of Figures 2 and 3 and listed in Tables S4 and S5, that is, compounds and proteins that showed a significant temperature effect at one temperature decrease at least (corrected *p*‐value < .01 and fold‐change >1.2 or <1.2^−1^). Their patterns of changes were visualized using K‐means clustering (Figure 4, Table S8). Cluster 1 comprises six metabolites and 42 proteins that decreased greatly from 20°C to 16°C and then remained stable or varied very slowly. It includes malate, three amino acids, six ribosomal proteins and nine proteins involved in porphyrin or chlorophyll metabolic processes. Cluster 2 comprises seven metabolites and 13 proteins that decreased from 20°C to 16°C and then increased slowly for at least one other temperature step. It includes aspartate, choline, HMBOA‐glucoside, DIM_2_BOA‐glucoside, two superoxide dismutases and four proteins involved in nitrogen metabolism. Cluster 3 comprises 13 metabolites and 22 proteins that increased from 20°C to 16°C and continued to increase slowly from 16°C to 8.5°C. It includes raffinose, sucrose and sucrose synthase, glycine, eight specialized metabolites, a glutamine synthetase, two glutathione transferases and early light‐induced protein. Cluster 6 comprises 10 compounds and 33 proteins that increased from 20°C to 16°C and then remained stable or decreased very slowly. It includes starch and starch synthase IIc precursor, *trans*‐aconitate, three proteins involved in lipid metabolic processes and five proteases. Cluster 4 comprises four metabolites and one protein that showed various abundance patterns between 20°C and 16°C, but all peaked at 13°C. It includes fructose and a protein involved in benzoxazinoid biosynthesis. Cluster 5 comprises one metabolite and seven proteins that decreased continuously from 20°C to 8.5°C. It includes serine/threonine‐protein kinase SNT7 and four enzymes involved in lipid metabolism.

**FIGURE 5:**
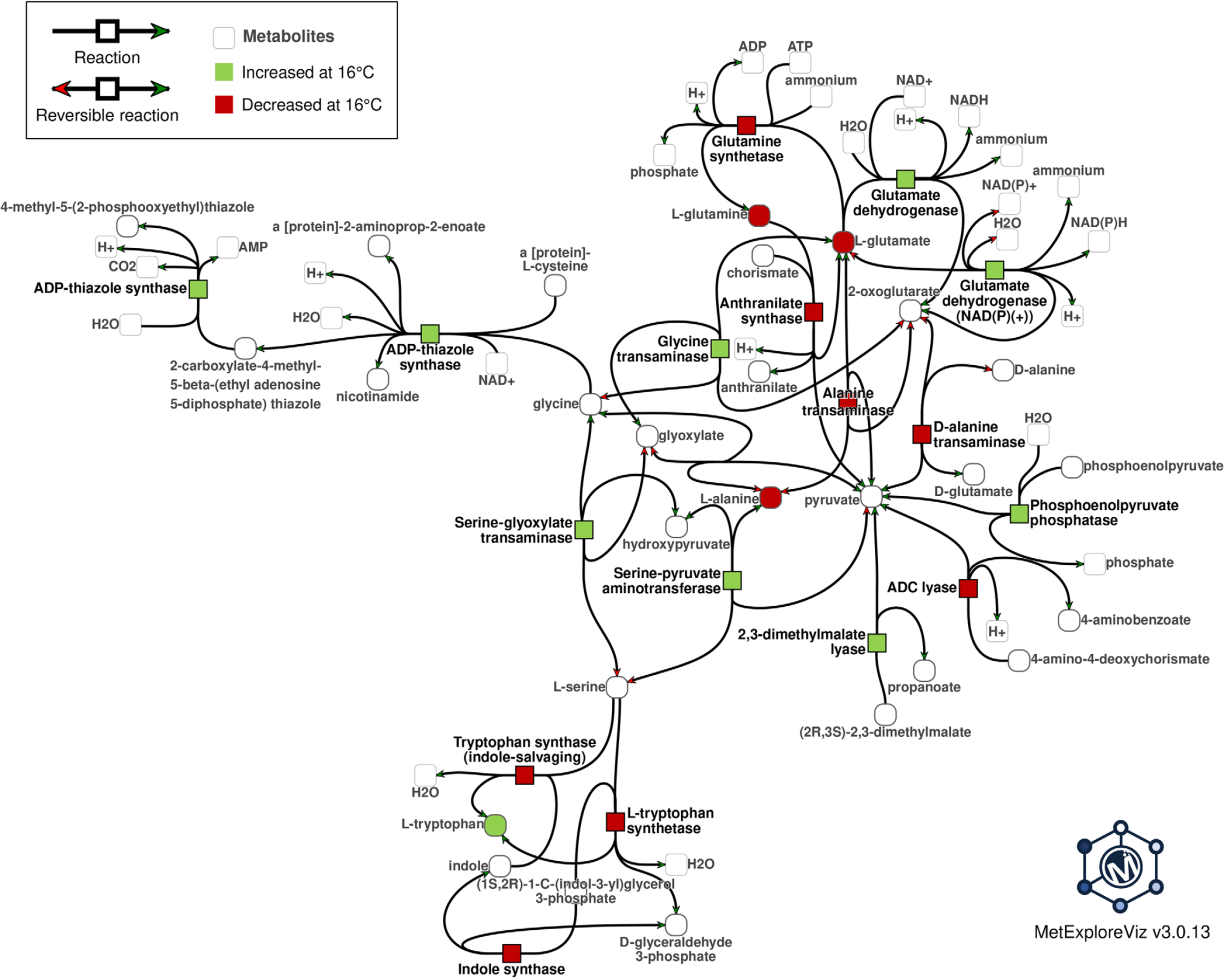
Example of significant metabolism changes induced by progressive decreasing temperatures in leaves of maize plants cultivated in growth chamber, according to volcano plot analyses of Figures 2 and 3. Annotated metabolites and enzymatic proteins up or down‐regulated after 2 days at 16°C are mapped on a genome‐wide metabolic network. The largest sub‐network is presented. Squares, proteins; circles, metabolites. Red, down‐regulation; green, upregulation [Colour figure can be viewed at wileyonlinelibrary.com]

The 29 compounds identified with a ChEBI ID and the 118 annotated proteins previously mentioned were mapped on genome‐wide metabolic pathways (Figure S7) for the 20°C to 16°C step when significant in the volcano plots. Figure S7 comprises 12 sub‐networks with more than two reactions including three large sub‐networks. One of these largest sub‐networks comprises enzymes involved in redox state regulation the majority of which are up‐regulated at 16°C. Another large sub‐network concerns photosynthetic pigment metabolism with a majority of enzymes down‐regulated at 16°C. Zooming in on the other largest sub‐network for this temperature change showed a decrease in alanine, glutamine and glutamate contents and an increase in two glutamate dehydrogenases as well as in several aminotransferases involved in photorespiration (Figure 5).

### 3.5 Responses to chilling in the growth chamber and in the field are similar for nine compounds and 71 proteins

**FIGURE 6:**
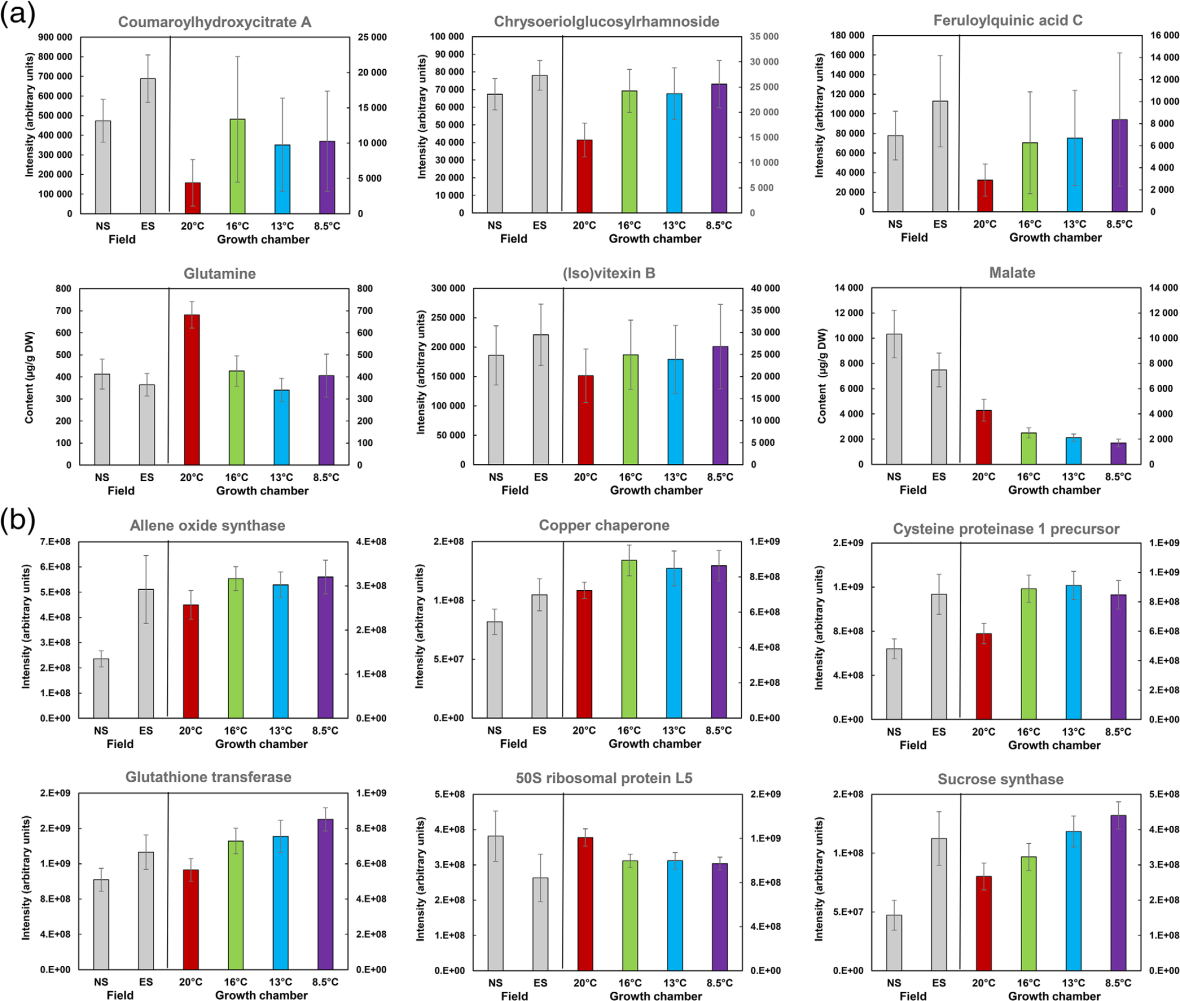
Comparison of leaf responses between growth‐chamber (*y*‐axis on the right hand‐side) and field experiments (*y*‐axis on the left hand‐side) for (a) a selection of six identified compounds and (b) six annotated proteins having the highest absolute values of loadings in Figure S8 PCA. Mean of the 16 maize hybrids common to the field and the growth‐chamber experiments. Vertical bars represent standard deviations [Colour figure can be viewed at wileyonlinelibrary.com]

Sixteen of the 18 genotypes cultivated in the growth chamber were also cultivated in the field and harvested for biochemical phenotyping, performed in other analytical batches. Plants were sown at two dates 28 days apart, and the mean air temperature of early‐sowing and normal‐sowing plants was 13.5°C and 15.6°C, respectively. To compare the effects of chilling in the field to those observed in the growth chamber, we analysed 26 compounds and 125 proteins that had shown a significant temperature effect in the growth‐chamber experiment and that could be measured in the field experiment: 26 compounds out of the 39 significant and annotated ones in Table S4, and 125 proteins out of the 214 significant proteins in Table S5). To visualize the changes, we performed PCAs on field compound and protein data separately. In both cases, early‐sowing and normal‐sowing samples were very well separated along PC1 (25% and 21% of total explained variance for compound and protein data, respectively, Figure S8). Among the 17 compounds (16 metabolites plus starch) that were up‐regulated by chilling in the growth chamber, seven followed similar patterns after early sowing in the field compared to normal‐sowing (based on the sign of their loadings, Figures S8a, b). These metabolites included *trans*‐aconitate, a coumaroylhydroxycitrate, chrysoeriolglucosylrhamnoside, a caffeoylquinate, a ferruloylquinate, an (iso)vitexin and DIBOA‐glucoside. Two (malate and glutamine) of the three metabolites that were down‐regulated by chilling in the growth chamber were also down‐regulated in response to early sowing in the field. The seven metabolites that followed non‐monotonous trends in the growth‐chamber experiment could hardly be compared between the two experiments. Among the 61 proteins that were up‐regulated by chilling in the growth chamber, 43 followed a similar pattern in response to early sowing (Figures S8c, d). These included a sucrose synthase, lethal leaf‐spot 1, a copper chaperone, a cysteine proteinase 1 precursor, an allene oxide synthase, a coumarate‐CoA ligase and several glutathione transferases and peroxidases. Among the 64 proteins that were down‐regulated by chilling in the growth chamber, 28 followed the same pattern after early sowing in the field compared to normal‐sowing. These included a 6‐phosphofructo‐kinase, a phosphomethylpyrimidine synthase, a magnesium chelatase and ribosomal protein L5. The six metabolites and six proteins with the highest absolute values of loadings in each PCA of field data in Figure S8 are presented in Figure 6, beside the corresponding data for the growth chamber for the 16 common genotypes. Interestingly, these six metabolites and six proteins followed three different patterns in the growth chamber (Figure 4 KMC analysis). Glutamine, malate and 50S ribosomal protein L5 belong to cluster 1. Chrysoeriolglucosylrhamnoside, feruloylquinate C, (iso)vitexin B, a glutathione transferase and sucrose synthase belong to cluster 3. Coumaroylhydroxycitrate A, an allene oxide synthase, a copper chaperone and cysteine proteinase 1 precursor belong to cluster 6. All these clusters show high changes between 20°C and 16°C: decreases for cluster 1 and increases for cluster 3 and 6.

### 3.6 Genotype groups based on field phenotype can be distinguished based on their metabolic responses in the growth chamber

Among the 18 hybrids of the growth‐chamber experiment, 16 were common with field experiments performed in 2013 (Lamari et al., 2018) and 2014 (present work), in which aerial biomass was measured for early‐ and normal‐sowing conditions. Mean field temperatures were below 16°C in both sowing conditions. We used these phenotypic data to classify the 16 hybrids into three groups according to their biomass ratio between early‐ and normal‐sowing conditions (Table S9). The B73 hybrid was classified in the group with the lowest biomass ratio, in line with a previous study where it had been described as “cold‐sensitive” (Revilla et al., 2014).

Using OSC‐PLS‐DA, we then sought to highlight the metabolic or protein responses (i.e., ratio between temperature steps) in the growth‐chamber conditions that may differentiate these three genotype groups. The metabolic and protein responses with the highest VIP scores (VIP scores >2 threshold) could give clues about the molecular profiles of ideotypes adapted or less affected by early sowing, that is, maintaining the highest biomass ratio between early‐ and normal‐sowing. Hybrids whose biomass was less affected by early sowing were characterized by higher ratios of sucrose, putrescine, glycine and tryptophan at 13 over 16°C, lower ratios of aspartate, glutamate, succinate, two caffeoylquinates and a caffeoylhydroxycitrate at 13 over 16°C, and lower ratios of acetate and coumaroylquinate at 8.5 over 13°C (Figures S9a, b). Regarding proteomic data, these hybrids were also characterized by higher ratios of nitrile‐glutathione‐R‐transferase at 13 over 16°C and of cysteine protease 1 at 8.5 over 13°C. They presented lower ratios of ferredoxin‐nitrite‐reductase, protochlorophyllide‐reductase B and kri1 ketol‐acid‐reductoisomerase 1 at 13 over 16°C, and lower ratios of small nuclear ribonucleoprotein‐associated protein B, glutathione‐transferase, early light‐induced protein and glutathione‐transferase 5 at 8.5 over 13°C (Figures S9c, d). These promising results should be confirmed using other field biomass data and compositional data of a larger genotype panel.

## 4 DISCUSSION

### 4.1 Metabolic responses to controlled short‐term chilling in maize involve regulation of protein synthesis and modulation of photosynthesis, primary and specialized metabolisms

Decreasing the temperature from 20°C to 16°C and below had a strong effect on several ribosomal proteins and on the total soluble protein content of maize leaf. Such a decrease is in line with the suppression of de novo accumulation of several abundant proteins shown in rice leaf under a cold treatment using in vivo labelling (Hahn & Walbot, 1989), and with the downregulation of several ribosomal proteins shown in cabbage leaf in response to low temperature (Yuan et al., 2019). Furthermore, we found that nitrate reductase and ferredoxin‐nitrite reductase protein relative contents were strongly decreased, in agreement with the decreased nitrogen assimilation observed in response to chilling in several species including maize (Schlüter et al., 2013).

The relative contents of proteins involved in several chloroplast functions, including photosynthetic pigment metabolism and photosynthetic electron transport chain, decreased with temperature. This trend is in line with the previously proposed role of various LHCII complexes in the organization of photosystem II and photoprotection in maize under low temperature (Caffarri, Frigerio, Olivieri, Righetti, & Bassi, 2005). The decrease in malate, the central metabolite in maize C4 photosynthesis, is in agreement with a decreased photosynthetic fixation of CO_2_ (Yamori, Hikosaka, & Way, 2014)_._ As malate is a redox equivalent carrier (Igamberdiev & Eprintsev, 2016), it may also be related to redox regulations. Several enzymes involved in photorespiration were up‐regulated when lowering the temperature from 20°C to 16°C. The decrease in the leaf content of glutamate and glutamine that contribute to the glutamate‐glutamine shuttle between mitochondria and plastids seems in line with a high photorespiration rate. Although low temperatures increase the affinity of Rubisco for CO_2_ relative to O_2_ and increase the solubility of CO_2_ (Usadel et al., 2008), which is crucial for C3‐photosynthesis plants, photorespiration is believed to contribute to mitigating oxidative stress under abiotic stress conditions, including chilling stress for C3‐ and C4‐photosynthesis plants (Hodges et al., 2016; Voss, Sunil, Scheibe, & Raghavendra, 2013). The metabolome data showed increased contents in sucrose, raffinose and starch with decreasing temperatures. Increases in raffinose and several other soluble sugars were also highlighted in a cold experiment on wheat in controlled conditions (Zhao et al., 2019).

Overall for primary metabolism, decreasing the temperature from 20°C to 16°C induced a regulation of photosynthesis, photorespiration and carbohydrate metabolism in global agreement with a recent review about plant metabolism during cold acclimation (Furtauer, Weiszmann, Weckwerth, & Nagele, 2019), more extensively studied in C3‐ than in C4‐photosynthesis plants. In the present experiment, cold also induced modifications of nitrogen assimilation (nitrate reductase), amino acid metabolism and protein synthesis.

For specialized metabolism, several phenylpropanoids or benzoxazinoids were affected. Four flavonoids, including maysin and nine hydroxycinnamic acids (a caffeoylhydroxycitrate, four caffeoylquinates, a coumaroylhydroxycitrate, a coumaroylquinate, a feruloylhydroxycitrate and a feruloylquinate) were increased at 16°C. Maysin has recently been shown to respond to a combination of abiotic and biotic stresses in maize leaf (Block et al., 2020). The increase in hydroxycinnamates, besides their potential antioxidant role, may be related to vegetative growth reduction under cold, as a coumaroylquinate and a caffeoylquinate in maize leaf were previously shown to be negatively correlated with yield‐related traits (Cañas et al., 2017). Four benzoxazinoids, usually involved in biotic stress responses, (DIMBOA‐glucoside, DIM_2_BOA‐glucoside, HBOA‐glucoside, HMBOA‐glucoside) decreased at 16°C and one increased (DIBOA‐glucoside). Such a response for an abiotic stress is in line with a transcriptomic study on several stresses in a maize genotype leaf (Li et al., 2017), showing that the benzoxazinoid pathway was significantly overrepresented among genes differentially expressed in response to drought and cold treatments.

Our results are in partial agreement with a proteomic study comparing maize seedlings of one line at 25°C and 4°C (Wang et al., 2016). Although the responses of individual proteins were not all similar in both studies, several common responses to low temperature include alleviation of photodamage, modified carbohydrate and nitrogen metabolism, increased abundance of stress‐responsive proteins, improvement in the ability to scavenge ROS (detoxifying enzymes and antioxidants), protein modifications and modified lipid metabolism.

### 4.2 Metabolic responses of maize and Arabidopsis to short‐term chilling are different

The response of maize to chilling was different from that obtained by Usadel et al. (2008), who analysed the response of Arabidopsis to slight temperature decreases (from 20 to 17, 14, 12 or 10°C) using a multi‐level approach including transcriptomics, metabolomics and analysis of enzyme activities. This is probably related to the fact that Arabidopsis is a chilling‐tolerant plant while maize is a plant of subtropical or tropical origin which has adapted to temperate conditions, thanks to long‐term breeding efforts (Swarts et al., 2017). One of the most striking differences is that the total leaf protein content increased in response to chilling in Arabidopsis while it decreased in maize. This was confirmed by the fact that the protein synthesis machinery was induced in Arabidopsis whereas it was altered in maize. Maize leaf thus appears unable to adjust its metabolism by increasing protein concentrations to compensate for the decrease in catalytic rates (Stitt & Hurry, 2002). Importantly, the amount of nitrate reductase, the first step in nitrogen assimilation, increased in Arabidopsis while it decreased dramatically in maize. Sugars accumulated in both species in response to chilling, but to a much lesser extent in maize. There was no switch from starch to sucrose in maize, as starch increased in maize while it decreased in Arabidopsis, indicating that maize leaf was less efficient in partitioning carbon for the purpose of cryoprotection. Finally, the most resistant maize hybrids (Group 3) accumulated more sucrose and maintained higher levels of ferredoxin‐nitrite reductase in response to chilling in the growth chamber. These responses suggest better cryoprotection and maintenance of nitrogen assimilation, which both certainly play an important role in the tolerance to chilling of Arabidopsis.

### 4.3 A SNT7 kinase and a geranylgeranyl hydrogenase respond to all temperature decreases

Only two proteins decreased at all temperature steps: an SNT7 serine/threonine kinase and a geranylgeranyl hydrogenase, both related to chlorophyll metabolism. SNT7 kinase plays multiple roles in cells. For instance, its phosphorylation of thylakoid proteins contributes to maintaining the structure of the photosynthetic machinery, balancing excitation between PSII and PSI, and signalling by controlling redox balance in the electron transfer chain (Tikkanen, Gollan, Suorsa, Kangasjarvi, & Aro, 2012). SNT7 kinase was shown to be up‐regulated in response to drought stress in wheat (Shao et al., 2018). Its downregulation in the present work, in parallel with the regulation of chlorophyll accumulation observed, may lead to an increase in the PSI‐to‐PSII ratio during thylakoid membrane biogenesis and contribute to a complex regulation of ROS signalling (Tikkanen et al., 2012). The geranylgeranyl hydrogenases are involved in various processes, including chlorophyll biosynthesis. The gene expression of a geranylgeranyl hydrogenase was shown to be up‐regulated in spinach leaf, a cold‐tolerant species, after a heat treatment (Yan et al., 2016) and down‐regulated in peach leaf by cold stress (Giannino et al., 2004).

### 4.4 Short‐term growth‐chamber chilling partly mimics field conditions

About half of the metabolites and proteins that were affected by chilling in the growth‐chamber conditions exhibited similar responses to cold stress in the field experiment, where stress was induced by early sowing. Shared responses included generic responses to stress and were related to the limitation of oxidative damage (increases of several glutathione transferases, peroxidases and phenolic compounds) or jasmonate signalling (increases in an allene oxide synthase [Farmer & Goossens, 2019]). They also concerned the compatible solute sucrose (increased sucrose synthase), and *trans*‐aconitate major organic acid in maize leaf (Cañas et al., 2017), also present in phloem sap (Yesbergenova‐Cuny et al., 2016), which increased with cold. The latter may also be a general response to stress, as an increase in *trans*‐aconitate has been observed in maize in response to drought (Sicher & Barnaby, 2012), and is possibly common to several cereals. *Trans‐*aconitate is formed from citrate by citrate dehydrase, or from *cis*‐aconitate (involved in the tricarboxylic acid [TCA] cycle) by aconitate isomerase. It represents an additional pool of fixed carbon that can be stored in vacuoles and may help to stabilize the TCA cycle (Igamberdiev & Eprintsev, 2016). In addition, the observed *trans*‐aconitate increase may be indirectly related to the decreased N assimilation (less need of anaplerotic replenishment of TCA cycle intermediates), evidenced by decreased glutamine contents. A transcriptome analysis of senescence in wheat leaf highlighted a cytoplasmic aconitate hydratase among the up‐regulated genes, in relation with nitrogen recycling (Gregersen & Holm, 2007). Increases in lethal leaf‐spot 1, a pheophorbide *a* oxygenase shown to be up‐regulated in response to wounding stress in maize (Yang, Wardzala, Johal, & Gray, 2004) and to regulate cell death and pathogenesis‐related genes in wheat (Tang et al., 2013), may be crucial to protect the photosynthetic apparatus from the phototoxic chlorophyll catabolite pheophorbide *a* (Pružinská, Tanner, Anders, Roca, & Hörtensteiner, 2003) under chilling.

The partial discrepancies observed between the controlled growth‐chamber and the field conditions are not surprising as the duration, temperatures and light conditions differed. Indeed, light intensity has been shown to be as important as temperature during cold acclimation of maize (Szalai et al., 2018). Importantly, mean field temperatures were below 16°C in both normal‐sowing and early‐sowing conditions, while most of the variations in metabolites and protein content occurred between 20°C and 16°C in the growth chamber. Therefore, the proteins and metabolites which varied in the field but remained consistent in the growth‐chamber experiment may participate in the long‐term adaptation to cold temperatures; those that showed no variation consistency or varied in the opposite direction may help short‐term adaptation. These various behaviours could be related to protein synthesis and degradation rates. For metabolite contents, several responses observed in the growth‐chamber experiment are in line with a previous maize field experiment in 2013, in which cold stress was induced by early sowing (Lamari et al., 2018). The progressive decreases in glutamate, malate and alanine across all temperature steps were also observed when comparing early‐ to normal‐sowing in the field. The progressive increases or plateauing in tryptophan, starch, shikimate and a coumaroylquinate across all temperature steps were also observed when comparing early and normal sowing in the field.

### 4.5 Metabolic response ideotypes could be useful for breeding

No genotype x temperature interactions were observed in the growth‐chamber experiment for the metabolome or proteome data. However, the part of variance explained by the genotype x temperature interaction was of the same order of magnitude as that explained by genotype. Based on the OSC‐PLS‐DA results, 20°C to 16°C responses did not contribute to differentiating the hybrid groups. However, responses to the lower temperature steps did, in line with the fact that mean temperatures in the field were below 16°C. Therefore, metabolic ideotypes could be proposed for adaptation to early sowing. Decreasing protochlorophyllide reductase B from 16°C to 13°C may be related to photosynthesis tuning in response to low‐temperature stress, allowing excess ROS to be scavenged (Gan, Liu, Li, Wang, & Luo, 2019). However, decreasing early light‐induced protein from 13°C to 8.5°C is surprising as it seems to be involved in protection against photooxidative stress in Arabidopsis (Hutin et al., 2003). For nitrogen metabolism, decreasing ferredoxin‐nitrite‐reductase, branched‐chain amino acid synthesis (kri1 ketol‐acid‐reductoisomerase 1) and aspartate and glutamate contents when shifting from 16°C to 13°C may indicate a possible adaptation to chilling by a modulation of the relations between carbon and nitrogen metabolisms (Toubiana et al., 2016).

When changing from 16°C to 13°C, decreasing succinate and caffeoylquinates and increasing sucrose, putrescine, glycine and tryptophan contents characterized adapting hybrids as well. Sucrose is an osmoregulator and a cryoprotectant. Its accumulation has been observed in response to cold in several crops and may also have a protective effect in chloroplasts (Liu, Zhou, Xiao, & Bao, 2018). Polyamines such as putrescine are involved in chilling tolerance in tobacco (Wang et al., 2019). Increasing glycine content may be linked to a complex regulation of photorespiration that contributes to abiotic stress response (Voss et al., 2013). Glutathione transferases are known to mediate abiotic stress tolerance through catalytic and non‐catalytic functions (Nianiou‐Obeidat et al., 2017). Glutathione *S*‐transferase expression has been shown to correlate with increased stress tolerance in wheat and barley (Gallé et al., 2009; Rezaei, Shobbar, Shahbazi, Abedini, & Zare, 2013). However, in maize leaf of adapting hybrids, redox metabolism seemed tuned differently with increasing nitrile‐glutathione transferase from 16°C to 13°C, and decreasing glutathione‐transferase 5 and another glutathione‐transferase from 13°C to 8.5°C. If a phenotyping scenario (e.g., observing compositional changes between 16°C and 13°C) had to be proposed for breeding for chilling adaptation, sucrose, putrescine, glycine and tryptophan seem interesting candidates to be validated in further experiments, especially because their absolute quantification measurements could be performed at high throughput and low cost in thousands of samples with robot‐based platforms (Zhang et al., 2015).

## 5 CONCLUSION

The present study investigated 18 maize genotypes presenting a wide genetic diversity. The metabolomic and proteomic characterization of leaves of the hybrids cultivated in controlled conditions revealed chilling responses common to all genotypes (partly differing from those of Arabidopsis), some of which were found again in a field experiment. It highlighted the metabolic behaviour of the most adapted hybrids and helped identify several candidate metabolites and proteins that could be tested as biomarkers (possibly as response ratios between temperature conditions) to predict the response to early sowing in the field from a growth‐chamber experiment. As proposed for breeding drought‐tolerant potato varieties (Haas et al., 2020), combining metabolic‐ and molecular‐based selection may be of interest for breeding new cold‐tolerant maize varieties.

## CONFLICT OF INTEREST

The authors declare no conflict of interest.

## AUTHOR CONTRIBUTIONS

Catherine Giauffret and Hélène Sellier designed and performed the growth‐chamber and field experiments, plant phenotyping, sampling of plant material and analysis of phenotypic data; Annick Moing, Yves Gibon, Maria Urrutia, Stéphane Bernillon, Catherine Deborde, Mickaël Maucourt, Patricia Ballias and Camille Bénard designed and performed the metabolome experiments; Michel Zivy, Mélisande Blein‐Nicolas and Thierry Balliau designed and performed the proteome experiment; Annick Moing, Maria Urrutia, Daniel Jacob and Sylvain Prigent analysed and mapped the data; Annick Moing wrote the first version of the article; Michel Zivy, Mélisande Blein‐Nicolas, Maria Urrutia, Yves Gibon, Sylvain Prigent, Catherine Giauffret and Stéphane Bernillon reviewed and edited the manuscript. All authors read and approved the final manuscript.

## Supporting information


**Figure S1.** Cumulative thermal times and mean air temperatures for the field‐cultivated maize plants, from emergence to leaf sample harvest in 2014.

**Figure S2.** Maize plant aspect before (20°C) and after last step of temperature treatment (8.5°C) in growth chamber. Representative images of aerial part for two stable and two unstable genotypes, according to their biomass evaluated in the field and to operating quantum yield of PSII measured in growth chamber.

**Figure S3.** Venn diagrams for two‐way ANOVAs, for effects of temperature step, genotype and their interaction, of compound (a) or protein data (b) of maize leaf of plants cultivated in growth chamber.

**Figure S4.** Distribution of variance explained by each factor (*R* ^2^ of factor effect) in two‐way ANOVAs, for effects of temperature step, genotype and their interaction, for leaf compound or proteome data of maize plants cultivated in growth chamber.

**Figure S5.** Classification of proteins affected in maize leaf by 20°C to 16°C decrease in growth chamber in MapMan categorical bins.

**Figure S6.** Volcano plot analyses of leaf proteome data expressed on a DW basis for three temperature‐steps for 18 maize hybrids cultivated in growth chamber (results and functional annotations are detailed in Table S7).

**Figure S7.** Mapping of metabolite and protein changes in leaves of maize plants cultivated in growth chamber, highlighted with volcano plot analyses (Tables S4 and S5), on genome‐wide metabolic networks with MetExplore tool, for 20°C to 16°C temperature decrease.

**Figure S8.** PCA for leaf metabolome and starch and proteome data of 16 maize genotypes cultivated in the field. PCAs are based on ls‐means per genotype × sowing condition of 26 compounds and 125 proteins that showed significant chilling effect in growth chamber (volcano plot analyses) and were measured in the field.

**Figure S9.** OSC‐PLS‐DA based on leaf metabolite and starch or protein responses measured in growth chamber between three decreasing temperature steps, in order to differentiate the three maize hybrid groups based on field biomass responses.

**Table S1.** Experimental design of the growth‐chamber chilling experiment.
**Table S2.** LC‐QTOF‐MS identified compounds in maize leaf extracts for the growth‐chamber experiment.
**Table S3.** Starch contents in maize leaf for four temperature conditions in growth chamber and two sowing conditions in field.
**Table S4.** Results of volcano plot analyses (Figure 2) for temperature steps for all metabolites or metabolite features and starch measured in leaves of maize hybrids cultivated in growth chamber.
**Table S5.** Results of volcano plot analyses (Figure 3) for temperature steps for all individual proteins measured in relative abundance, using LC–MS/MS in young leaves of maize hybrids cultivated in growth chamber.
**Table S6.** Tentative annotation of eight MS‐based metabolic markers having same trend in maize leaf for all three temperature decreases in growth chamber.
**Table S7.** Results of volcano plot analyses (Figure S6) for temperature steps for all individual proteins expressed in absolute content on a DW basis, using LC–MS/MS in young leaves of maize hybrids cultivated in growth chamber.
**Table S8.** Detailed results of K‐means clustering analysis, presented in Figure 4, of 41 compounds and 118 proteins measured in leaves of maize hybrids cultivated in growth chamber.
**Table S9.** Classification of maize genotypes according to their aerial biomasses in the field after early sowing over 2 years.

## Data Availability

The metabolome ^1^H‐NMR spectra and LC‐MS files are available at https://data.inrae.fr/dataset.xhtml?persistentId=doi:10.15454/F2CZ1R and https://data.inrae.fr/dataset.xhtml?persistentId=doi:10.15454/J9KO72, respectively. The mass spectrometry proteomics data are available at ProteomeXchange (http://proteomecentral.proteomexchange.org/, dataset identifiers PXD021790 and PXD021754).
