# Supplementary figures and images for: Maize metabolome and proteome responses to controlled cold stress partly mimic early‐sowing effects in the field and differ from those of Arabidopsis
Source: Plant Cell Environ. 2021 Jan 25;44(5):1504–21. doi: 10.1111/pce.13993 (PMC8248070; doi:10.1111/pce.13993)

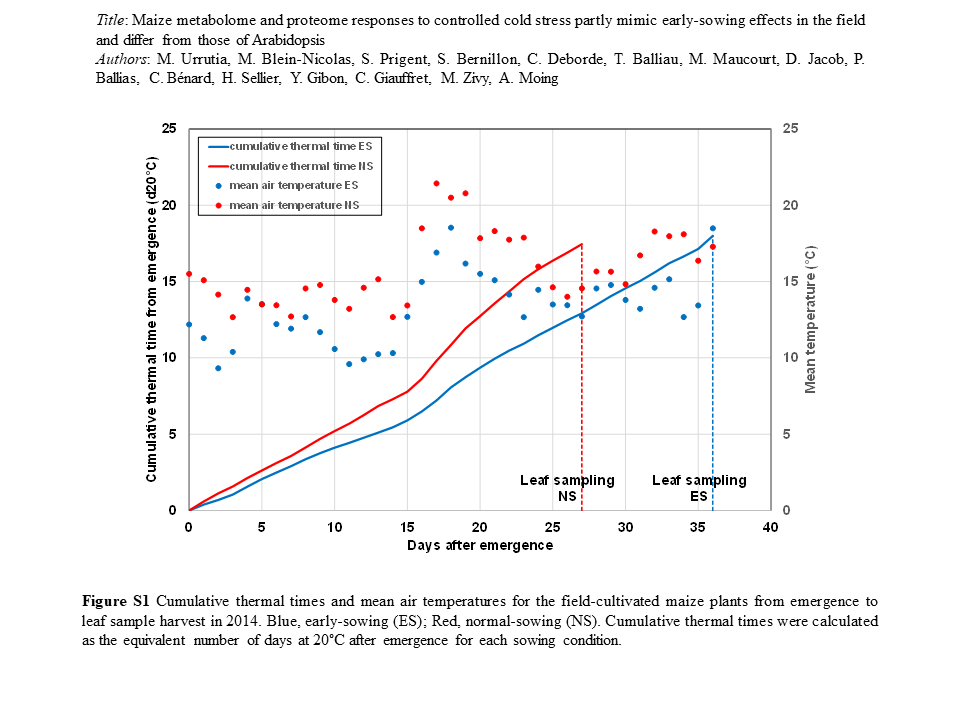

Supplement: Supplementary file 1 — Figure S1. Cumulative thermal times and mean air temperatures for the field‐cultivated maize plants, from emergence to leaf sample harvest in 2014. [file PCE-44-1504-s002.png]

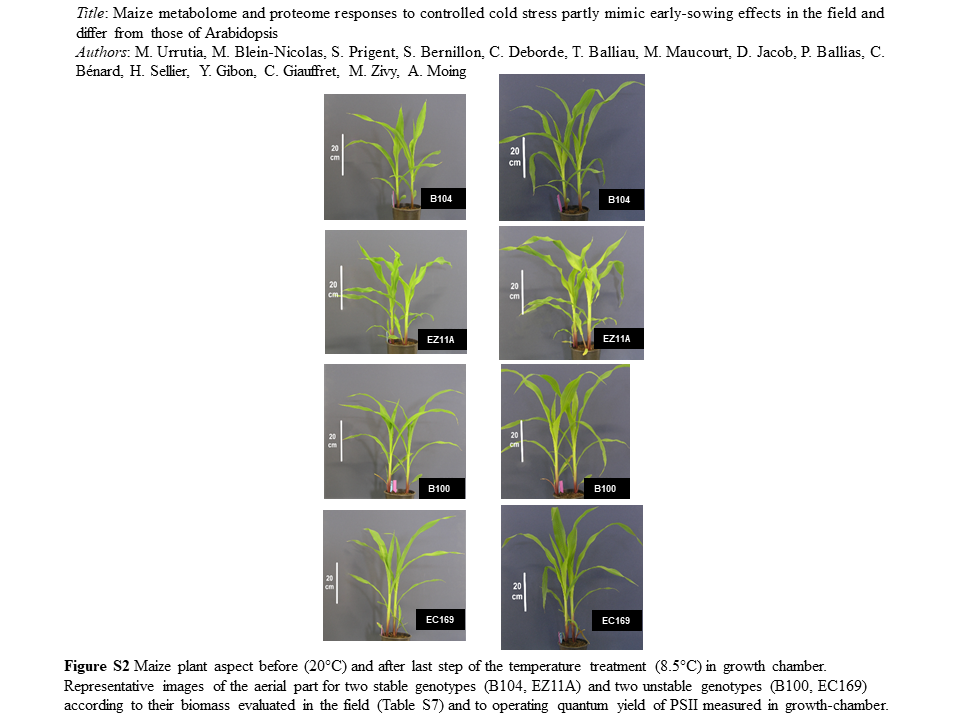

Supplement: Supplementary file 2 — Figure S2. Maize plant aspect before (20°C) and after last step of temperature treatment (8.5°C) in growth chamber. Representative images of aerial part for two stable and two unstable genotypes, according to their biomass evaluated in the field and to operating quantum yield of PSII measured in growth chamber. [file PCE-44-1504-s003.png]

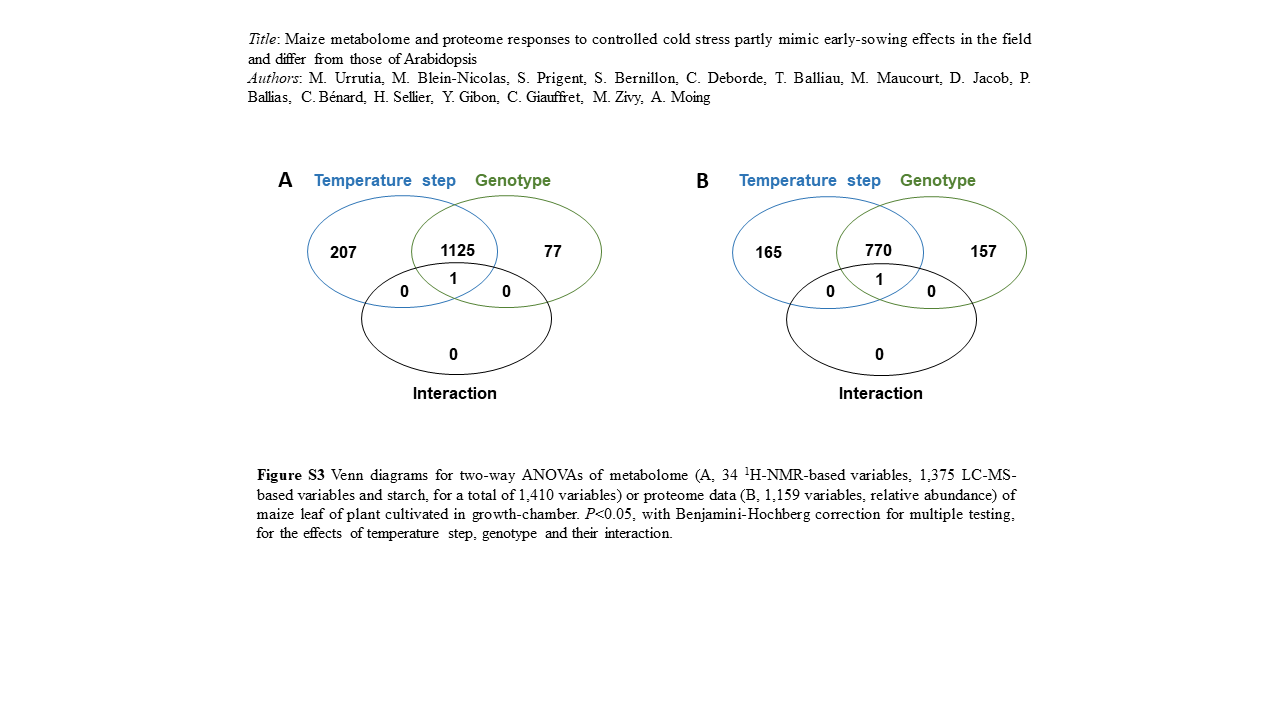

Supplement: Supplementary file 3 — Figure S3. Venn diagrams for two‐way ANOVAs, for effects of temperature step, genotype and their interaction, of compound (a) or protein data (b) of maize leaf of plants cultivated in growth chamber. [file PCE-44-1504-s006.png]

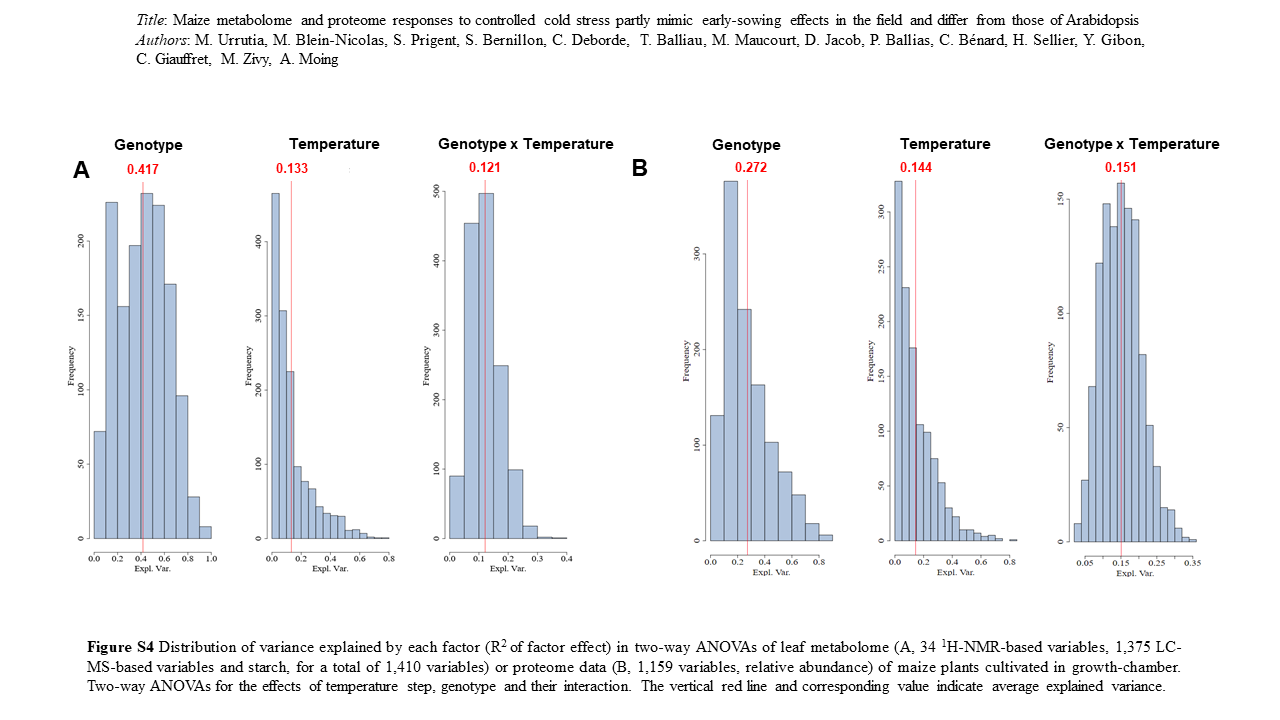

Supplement: Supplementary file 4 — Figure S4. Distribution of variance explained by each factor (R 2 of factor effect) in two‐way ANOVAs, for effects of temperature step, genotype and their interaction, for leaf compound or proteome data of maize plants cultivated in growth chamber. [file PCE-44-1504-s004.png]

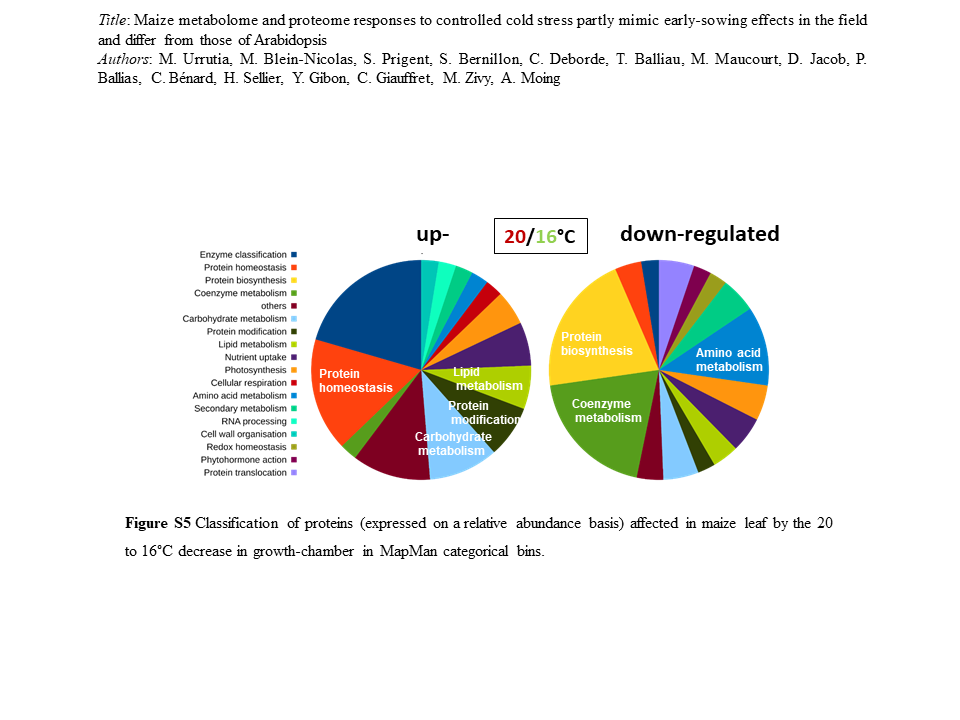

Supplement: Supplementary file 5 — Figure S5. Classification of proteins affected in maize leaf by 20°C to 16°C decrease in growth chamber in MapMan categorical bins. [file PCE-44-1504-s009.png]

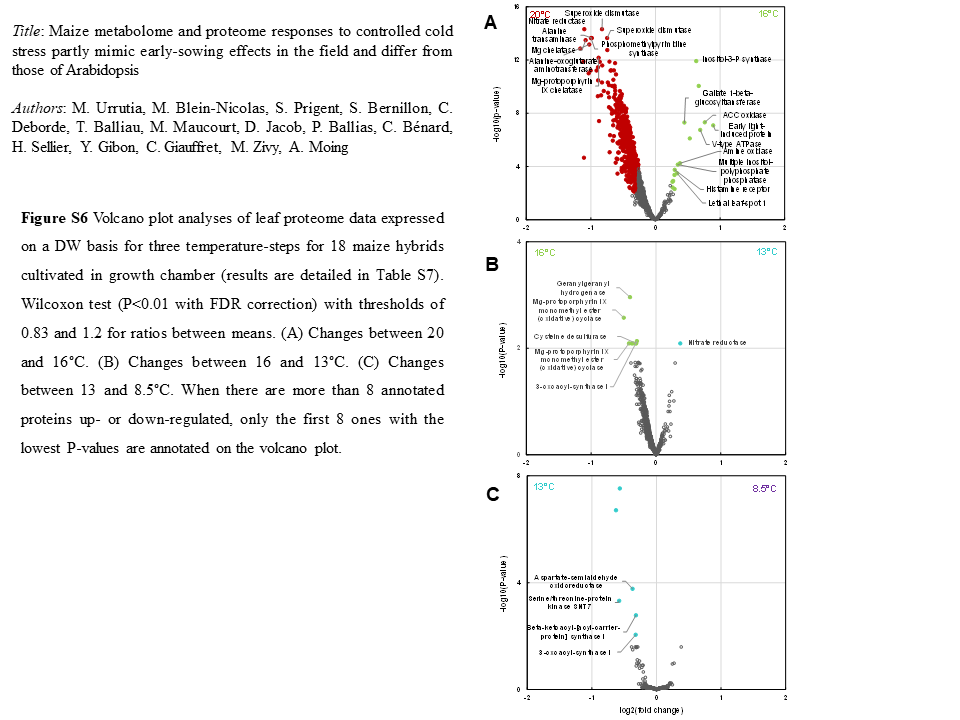

Supplement: Supplementary file 6 — Figure S6. Volcano plot analyses of leaf proteome data expressed on a DW basis for three temperature‐steps for 18 maize hybrids cultivated in growth chamber (results and functional annotations are detailed in Table S7). [file PCE-44-1504-s008.png]

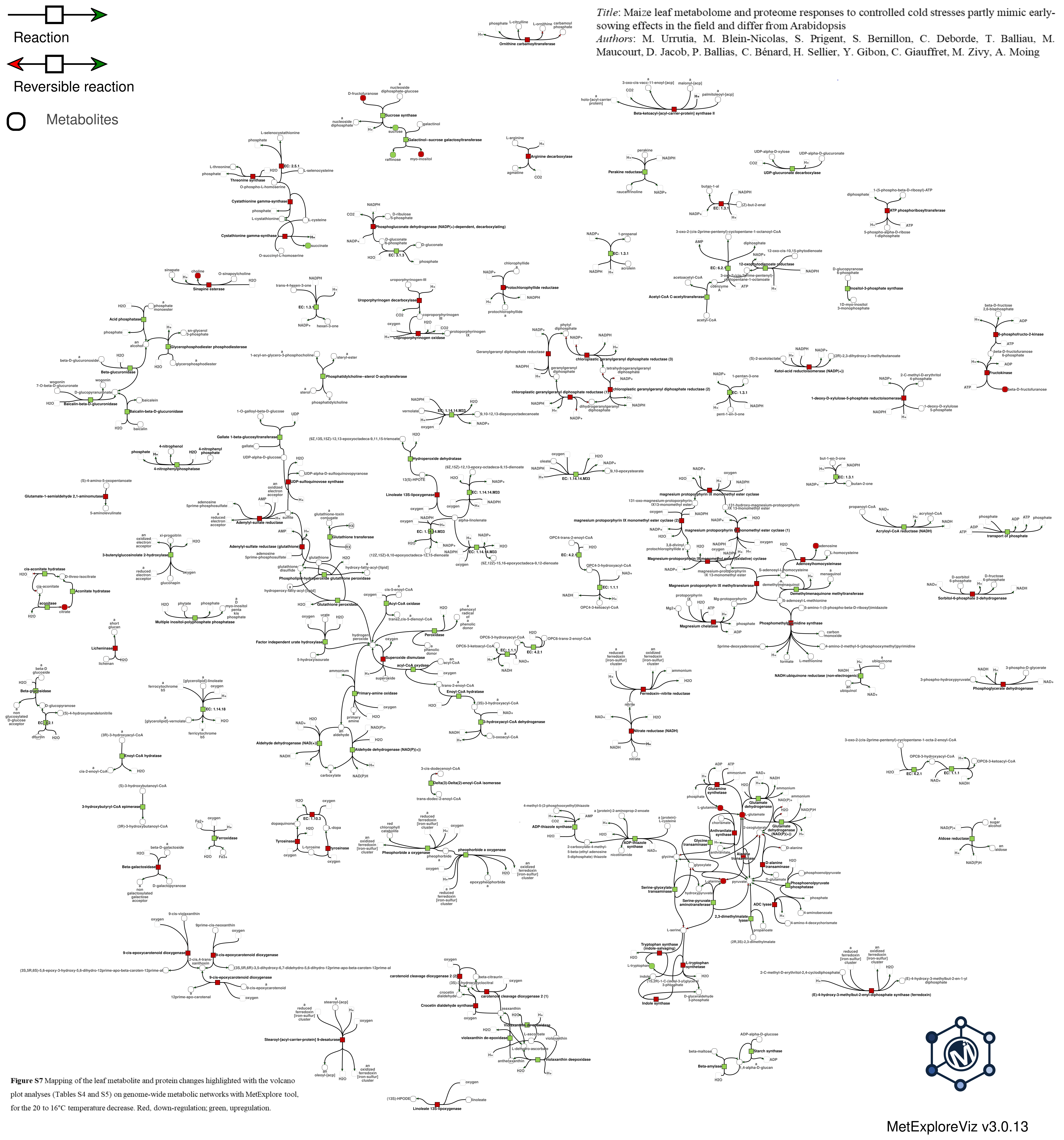

Supplement: Supplementary file 7 — Figure S7. Mapping of metabolite and protein changes in leaves of maize plants cultivated in growth chamber, highlighted with volcano plot analyses (Tables S4 and S5), on genome‐wide metabolic networks with MetExplore tool, for 20°C to 16°C temperature decrease. [file PCE-44-1504-s010.png]

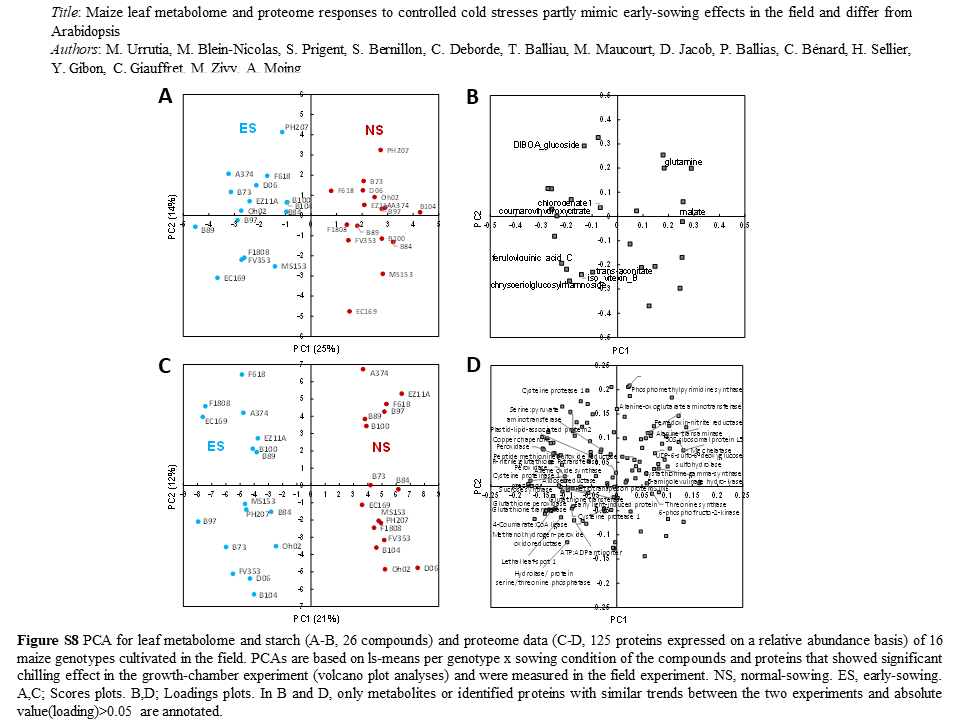

Supplement: Supplementary file 8 — Figure S8. PCA for leaf metabolome and starch and proteome data of 16 maize genotypes cultivated in the field. PCAs are based on ls‐means per genotype × sowing condition of 26 compounds and 125 proteins that showed significant chilling effect in growth chamber (volcano plot analyses) and were measured in the field. [file PCE-44-1504-s007.png]

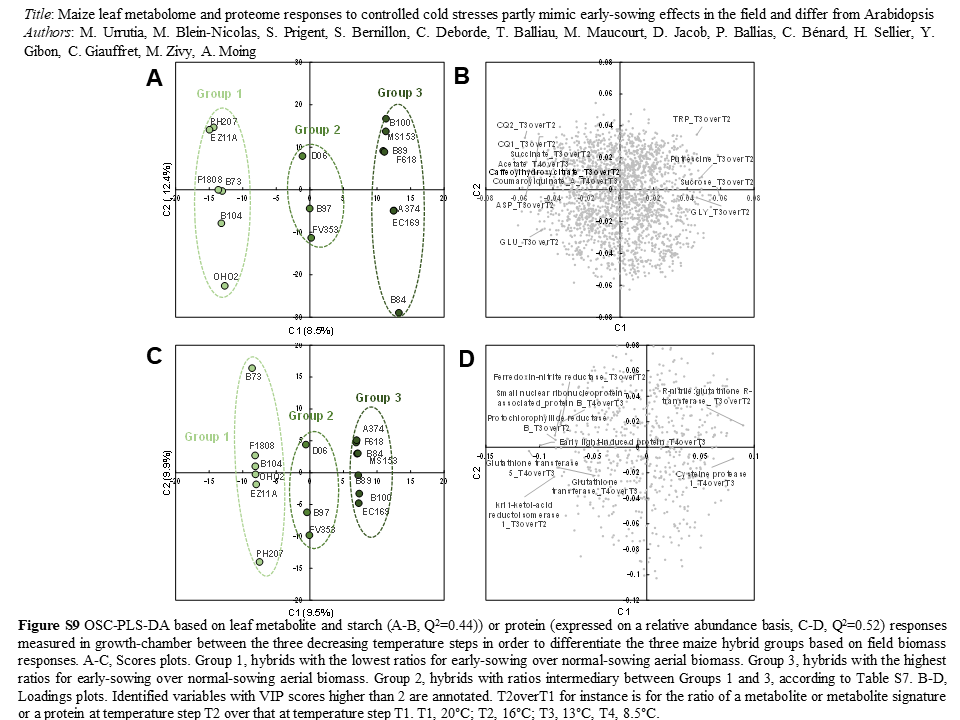

Supplement: Supplementary file 9 — Figure S9. OSC‐PLS‐DA based on leaf metabolite and starch or protein responses measured in growth chamber between three decreasing temperature steps, in order to differentiate the three maize hybrid groups based on field biomass responses. [file PCE-44-1504-s005.png]
